# The effects of intravenous iron supplementation on fatigue and general health in non-anemic blood donors with iron deficiency: a randomized placebo-controlled superiority trial

**DOI:** 10.1038/s41598-020-71048-0

**Published:** 2020-08-26

**Authors:** Peter Keller, Roland von Känel, Cesar A. Hincapié, Bruno R. da Costa, Peter Jüni, Tobias E. Erlanger, Nicola Andina, Christoph Niederhauser, Bernhard Lämmle, Stefano Fontana

**Affiliations:** 1 Division of Hematology, Department of Internal Medicine, SRO AG Spital Langenthal, Langenthal, Switzerland; 2 Department of Consultation-Liaison Psychiatry and Psychosomatic Medicine, University Hospital Zurich, University of Zurich, Culmannstrasse 8, 8091 Zurich, Switzerland; 3 Applied Health Research Centre (AHRC), Li Ka Shing Knowledge Institute, St. Michael’s Hospital, Toronto, Canada; 4 Department of Chiropractic Medicine, Faculty of Medicine, Balgrist University Hospital, University of Zurich, Zurich, Switzerland; 5 Institute of Health Policy, Management and Evaluation, Dalla Lana School of Public Health, University of Toronto, Toronto, Canada; 6 Clinical Trial Unit, Department of Clinical Research, University Hospital Basel, University of Basel, Basel, Switzerland; 7 Department of Hematology and Central Hematology Laboratory, Inselspital, Bern University Hospital, University of Bern, Bern, Switzerland; 8 Department for BioMedical Research, University of Bern, Bern, Switzerland; 9 Department of Biomedicine, University Hospital Basel, Basel, Switzerland; 10 Interregional Blood Transfusion SRC, Bern, Switzerland; 11 University of Lausanne, Lausanne, Switzerland; 12 Institute for Infectious Diseases, University of Bern, Bern, Switzerland; 13 Faculty of Biology and Medicine, Lausanne University Hospital, Lausanne, Switzerland; 14 Center for Thrombosis and Hemostasis, University Medical Center Mainz, Mainz, Germany; 15 Haemostasis Research Unit, University College London, London, UK

**Keywords:** Anaemia, Haematological diseases, Fatigue

## Abstract

We investigated whether intravenous iron supplementation improves fatigue and general health in non-anemic repeat adult blood donors with iron deficiency (ferritin ≤ 50 µg/L). Of 1,487 potentially eligible participants, 203 were randomly assigned to a single intravenous dose of 800 mg iron-carboxymaltose and 202 to placebo; 393 participants completed the trial. At 6 to 8 weeks after intervention, self-rated mean fatigue scores (numeric rating scale from 1–10, primary outcome) were 3.9 ± 1.8 in the iron supplementation group and 4.0 ± 2.2 in the placebo group, showing no group difference (*p* = 0.819). Pre-specified subgroup analyses of gender, ferritin < 25 µg/L and fatigue ≥ 4 points, as well as exploratory analyses of lower ferritin cut-offs did not reveal any between-group differences. In terms of secondary outcomes, the mean differences were 114.2 µg/L for ferritin (95% CI 103.1–125.3) and 5.7 g/L for hemoglobin (95% CI 4.3–7.2) with significantly higher values in the iron supplementation group. No group differences were observed for different measures of general well-being and other clinical and safety outcomes. Intravenous iron supplementation compared with placebo resulted in increase of ferritin and hemoglobin levels in repeat blood donors with low iron stores, yet had no effect on fatigue and general well-being.

## Introduction

Each year, about 120 million blood donations are provided by tens of millions of blood donors around the world^1^, enabling lifesaving transfusions and creating an invaluable resource for modern health systems. The safety and health of blood donors are therefore of great importance, and a better understanding of the health implications of blood donation is needed. Blood donation is associated with the loss of iron—every whole blood donation removes about 200–250 mg of elemental iron from the body^2^. Consequently, the iron stores of many repeat blood donors are low or depleted^3,4^. Among frequent donors in one US study, almost 20% and 30% of men and women, respectively, were found to have absent iron stores (ferritin < 12 µg/L)^5^. To prevent blood donation deferral due to iron deficiency anemia, measures to detect and correct low iron stores, including regular assessment of serum ferritin or iron supplementation^6–14^, have been proposed as routine measures by blood bankers^15–17^. Currently, however, iron supplementation for blood donors is not a standard of care in many blood donation services.

A syndrome of non-anemic iron deficiency (NAID) has been hypothesised as a potential factor leading to symptoms in the absence of anemia, especially given the essential role of iron in many metabolic pathways (e.g., mitochondrial oxidases^18^, neurotransmitters, muscle metabolism, and others^19^). NAID has been associated with fatigue^20–22^, reduced quality of life^23^, lower work performance^24^, cognitive changes^25–27^, mood disturbances^20,28^, and restless legs syndrome and pica^29–31^. However, few randomized clinical trials have examined participant reported outcomes, such as fatigue and general well-being, among blood donors receiving iron supplementation. In a Cochrane review^10^, only two trials were found which had included health-related quality of life outcome measures in blood donors receiving iron supplementation^32,33^.

We designed the Iron SUpplementation in Blood donors (ISUB) randomized controlled trial to assess the efficacy and safety of iron supplementation with intravenous ferric carboxymaltose in blood donors with NAID, focusing on participant reported outcomes of fatigue and well-being. In particular, we hypothesised a state of potentially impaired general well-being and increased fatigue due to NAID in repeat blood donors that might improve by intravenous iron supplementation. We used intravenous iron supplementation instead of oral supplementation to ensure sufficient and rapid iron availability, as well as true blinding, as oral iron formulations may cause gastrointestinal side effects, potentially impairing treatment adherence, and lead to a black discoloration of stool.

## Results

### Enrolment and follow-up

**Figure 1:**
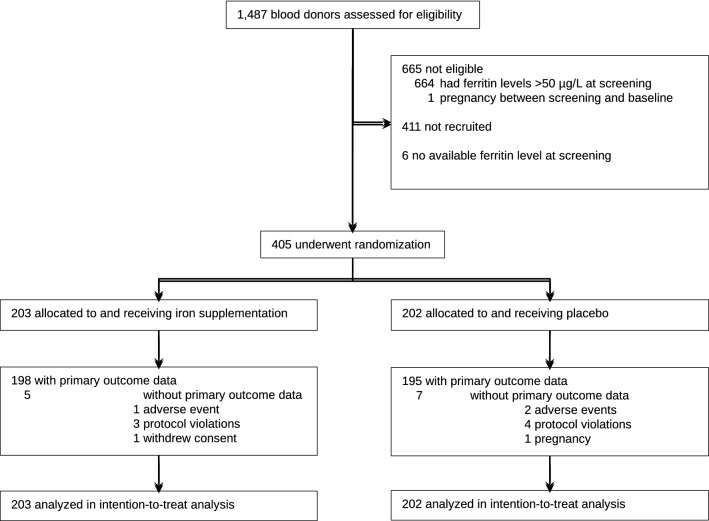
The figure shows the flow of participants through the trial.

Between December 6, 2011 and January 29, 2013, 405 participants underwent randomization: 203 were allocated to the experimental intervention and 202 to placebo (Fig. 1). One participant in the experimental group withdrew consent. Three and 4 participants had a protocol violation, and 198 (97.5%) and 195 participants (96.5%) provided primary outcome data at 6–8 weeks after randomization in experimental and control groups, respectively. All randomized patients were included in the intention-to-treat analysis. Baseline characteristics were similar in the two study groups (Table 1). The mean age was 42.2 years, the proportion of females 46.2%, and the mean number of blood donations over the past 2 years was 5. Mean hemoglobin on the day of treatment was 141.5 g/L in men and 125.0 g/L in women. We screened for iron deficiency 4 to 6 weeks prior to study enrolment on the occasion of a blood donation; the mean serum ferritin at this time point was 28.1 µg/L, while it was 18.6 µg/L on the day of randomization and treatment. At the time of iron or placebo supplementation, 176 (43.5%) blood donors had severe iron deficiency defined by ferritin < 15 µg/L.

**Table 1:** Baseline characteristics.

Characteristics	Iron group (N = 203)	Placebo group (N = 202)
Female—no. (%)	94 (46.3)	93 (46.0)
Age—year	42.1 ± 12.2	42.2 ± 12.3
Blood donations in the past 2 years	5.0 ± 1.9	5.3 ± 1.8
**Fatigue level**^**a**^	4.4 ± 2	4.6 ± 2
Fatigue level ≥ 4—no. (%)	116 (57.1)	116 (57.4)
**Multidimensional fatigue symptom inventory**^**b**^**—total**	16.5 ± 15	16.1 ± 15
MFSI—general	10.7 ± 4	10.5 ± 4
MFSI—physical	8.3 ± 3	8.2 ± 3
MFSI—emotional	9.4 ± 3	9.0 ± 3
MFSI—mental	9.4 ± 3	9.4 ± 3
MFSI—vigour	21.5 ± 4	21.2 ± 4
EQ-5D^c^ index	9.2 ± 1	9.3 ± 1
EQ-5D visual analog scale	8.3 ± 1	8.4 ± 1
27-item symptom checklist^d^ (SCL27)	1.3 ± 0	1.3 ± 0
Jenkins Sleep Scale^e^	9.6 ± 3	9.0 ± 3
Serum ferritin concentration at screening—µg/L	28.0 ± 12.2	28.2 ± 11.8
**Serum ferritin concentration at baseline—µg/L**	18.5 ± 9.8	18.7 ± 10.4
Ferritin < 25 µg/L at baseline—no. (%)	165 (81.3)	154 (76.6)
Ferritin < 15 µg/L at baseline—no. (%)	89 (43.8)	87 (43.3)
**Hemoglobin concentration—g/L**	133.8 ± 14.6	134.0 ± 12.6
Hemoglobin in males—g/L	142.5 ± 10.2	140.5 ± 10.4
Hemoglobin in females—g/L	123.6 ± 12.1	126.4 ± 10.5

Plus–minus values are means ± SD. There were no significant differences in baseline characteristics between the two randomly assigned groups. Percentages may not total 100 because of rounding

^a^Self-rated fatigue during the past 7 days on a numeric rating scale ranging from 1 (no fatigue at all) to 10 (extreme fatigue).

^b^30-item Multidimensional Fatigue Symptom Inventory-Short-Form (MFSI) measures general, physical, emotional, and mental fatigue, and vigour in the previous seven days. Each subscale comprises six items rated on a 5-point Likert scale from 0 (not at all) to 4 (extremely), yielding a total score between 0 and 24 for each subscale Total fatigue score is calculated by subtracting the vigour subscale score from the sum score of the other four subscales.

^c^EQ-5D is a generic instrument used to measure current health status related to mobility, self-care, usual activities, pain/discomfort and anxiety/depression. Each dimension is rated on three levels of perceived problems (with corresponding score): no problems (2), some or moderate problems (1), extreme problems (0), yielding a total standardized score between 0 and 10. Higher scores indicate better health status. The EQ-5D VAS allowed participants to rate their own health, ranging from 0 (worst imaginable health state) to 10 (best imaginable health state).

^d^27-item Symptom Checklist (SCL27) assesses general psychological distress based on depressive, anxiety, mistrust and vegetative symptoms in the previous week. Each item is rated on a 5-point Likert scale from 0 (not at all) to 4 (extremely). Total distress scores are expressed as the mean value of the 27 items.

^e^4-item Jenkins Sleep Questionnaire (JSQ) rates subjective sleep quality in the previous month, referring to trouble falling asleep, trouble staying asleep (waking up far too soon and inability to fall back to sleep), waking up several times per night, and waking up feeling tired and worn out after the usual amount of sleep. Response options (with corresponding score): not at all (0), 1–3 days (1), 4–7 days (2), 8–14 days (3), 15–21 days (4) and 22–31 days (5). Yields a total score between 0 to 20, with higher scores indicating poorer sleep quality.

### Outcomes

**Figure 2:**
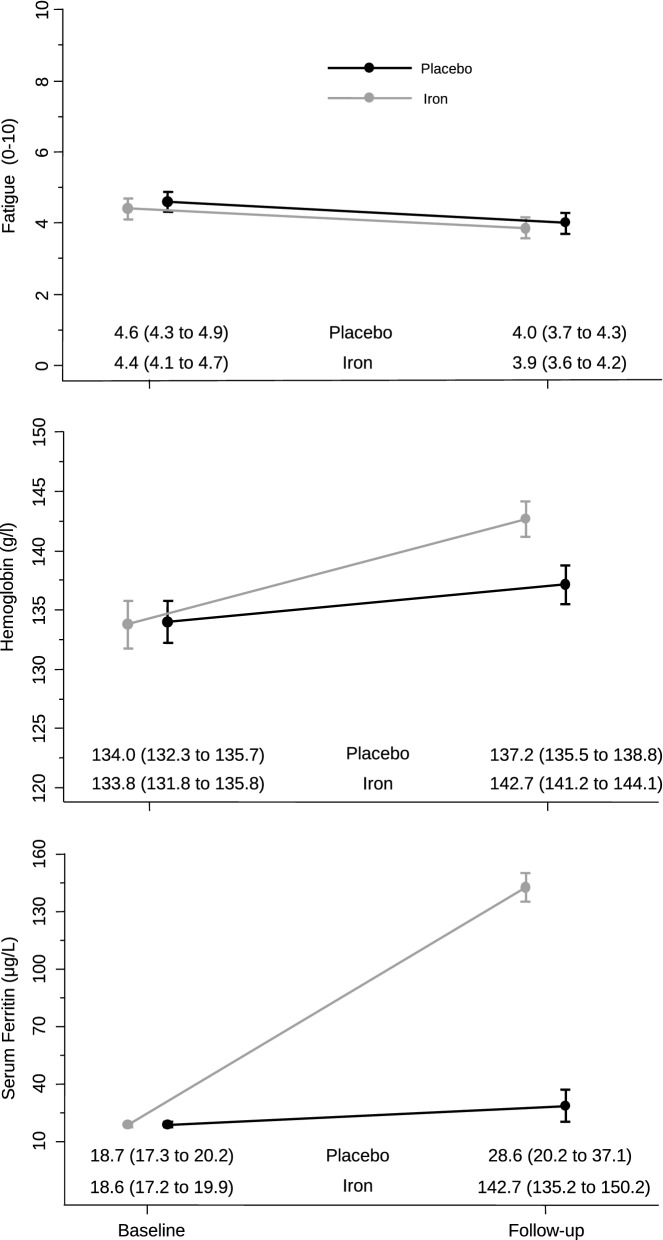
Self-rated fatigue, hemoglobin and ferritin levels at baseline and follow-up (6–8 weeks after randomization) in the iron and placebo groups. Data points are means; bars indicate 95% confidence intervals.

**Figure 3:**
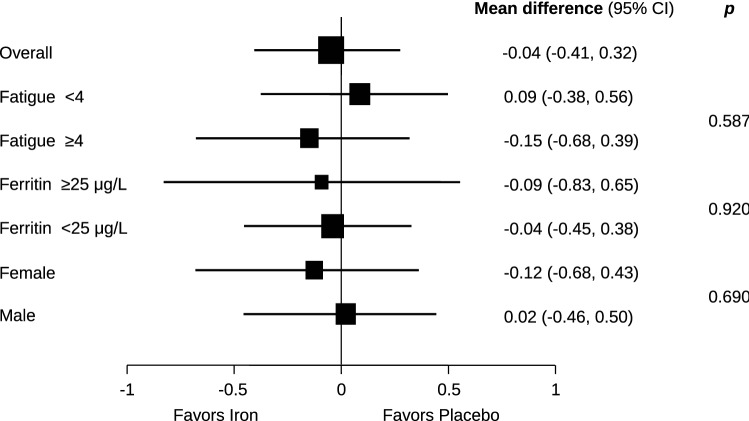
Forest plot of the primary outcome analysis for self-rated fatigue (numerical rating scale of 1–10 points) 6–8 weeks after randomization (overall) and the prespecified subgroup analyses according to baseline fatigue (< 4 vs. ≥ 4 points), ferritin level (< 25 µg/L vs. ≥ 25 µg/L), and gender. Mean differences with 95% confidence intervals are depicted. *p* values are for tests of interaction between the two treatment groups.

**Figure 4:**
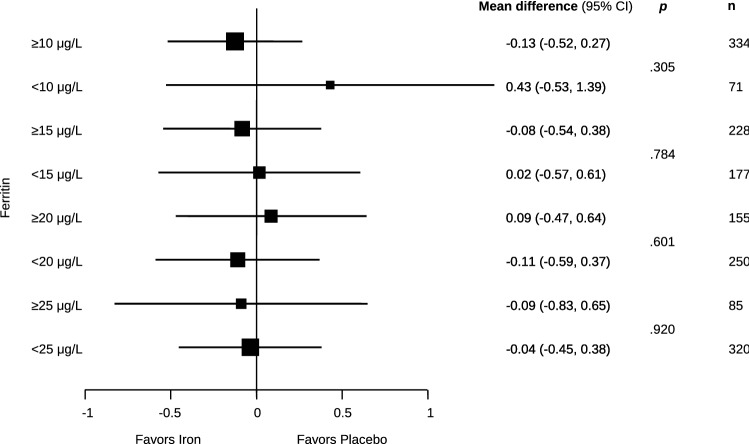
Forest plot of self-rated fatigue at follow-up for lower serum ferritin concentration level cut-offs of < 25, < 20, < 15 and < 10 µg/L. Shown are mean differences between iron supplementation and placebo groups with 95% confidence intervals and number of participants per subgroup. *p* values are for tests of interaction between the two treatment groups.

The mean values of the primary outcome of self-rated fatigue and all secondary outcomes are presented in Table 2, separately for the iron supplementation group and the placebo group, and with mean differences between groups. At 6 to 8 weeks after treatment, self-rated mean fatigue scores were 3.9 ± 1.8 in the iron supplementation group and 4.0 ± 2.2 in the placebo group, showing no group difference (mean difference − 0.04; 95% CI − 0.41 to 0.32; *p* = 0.813) (Fig. 2). Results were consistent for the other secondary donor reported outcomes at 6–8 weeks with no significant group differences. At 6–8 weeks the experimental group experienced a significantly larger increase in both serum ferritin (142.6 ± 54.5 µg/L vs. 28.6 ± 61.3 µg/L) and hemoglobin (142.7 ± 10.9 g/L vs. 137.2 ± 12.0 g/L) than the placebo group (mean differences 114.2 µg/L; 95% CI 103.1–125.3; *p* < 0.0001 for ferritin and 5.7 g/L; 95% CI 4.3–7.2; *p* < 0.0001 for hemoglobin) (Fig. 2). Pre-specified subgroup analyses of the primary outcome, including influence of gender (Fig. 3), of ferritin levels < 25 µg/L (Fig. 4) or of fatigue scores ≥ 4 (Fig. 3) did not show significant interactions with treatment. In addition, an exploratory subgroup analysis based on serum ferritin levels < 20 µg/L, < 15 µg/L and < 10 µg/L did not show a relevant impact on estimated treatment effects (Fig. 4).

**Table 2:** Primary and secondary outcomes.

Outcome	Iron group (N = 203)	Placebo group (N = 202)	Mean difference (95% CI)
**Primary outcome**			
Fatigue level	3.9 ± 1.8	4.0 ± 2.2	0.0 (− 0.4 to 0.3)
**Secondary outcomes**			
Change in fatigue^a^	− 0.6 ± 0.7	− 0.8 ± 2.2	0.1 (− 0.3 to 0.6)
MFSI—total	11.0 ± 12.0	11.3 ± 12.3	− 0.4 (− 2.3 to 1.5)
MFSI—general	9.2 ± 3.3	9.3 ± 4.0	− 0.1 (− 0.7 to 0.5)
MFSI—physical	7.3 ± 2.2	7.3 ± 1.8	0.0 (− 0.3 to 0.3)
MFSI—emotional	8.3 ± 2.9	8.1 ± 2.9	0.1 (− 0.4 to 0.6)
MFSI—mental	8.6 ± 2.5	8.5 ± 2.5	0.2 (− 0.2 to 0.6)
MFSI—vigour	22.4 ± 4.0	22.1 ± 4.7	0.0 (− 0.7 to 0.8)
EQ-5D index	9.5 ± 1.1	9.6 ± 1.1	− 0.1 (− 0.3 to 0.1)
EQ-5D visual analog scale	8.5 ± 1.1	8.4 ± 1.1	0.1 (− 0.1 to 0.3)
27-item symptom checklist (SCL27)	1.2 ± 0.0	1.2 ± 0.4	0.0 (− 0.0 to 0.0)
Jenkins sleep scale	8.5 ± 3.3	8.3 ± 2.9	− 0.2 (− 0.6 to 0.3)
Serum ferritin concentration—µg/L	142.6 ± 54.5	28.6 ± 61.3	114.2 (103.1 to 125.3)
Hemoglobin concentration—g/L	142.7 ± 10.9	137.2 ± 12.0	5.7 (4.3 to 7.2)

Plus–minus values are means ± SD. Continuous outcomes were analysed using analyses of covariance adjusted for the outcome’s baseline values and stratification variables. See legend to Table 1 for explanations of abbreviations.

^a^Self-perceived change in fatigue between baseline and 6–8 weeks after randomization, assessed on a NRS between − 10 and + 10.

### Safety outcomes

Table 3 presents adverse events. The total number of adverse events was greater in the iron supplementation group (n = 52) than in the placebo group (n = 29) (*p* = 0·019). Forty-one (20.2%) and 28 participants (13.9%) reported an adverse event in the experimental and placebo groups, respectively (*p* = 0·090). In each group, we observed one grade-3 adverse event: 1 participant reported dizziness and headache after iron supplementation, and 1 participant experienced retinal detachment after receiving placebo. There were no serious adverse events or suspected unexpected severe adverse reactions. Events certainly or likely associated with treatment were not significantly different between the two groups.

**Table 3:** Adverse events.

Event	Iron (N = 203)	Placebo (N = 202)	*p* value
**All adverse events**			
Number of participants reporting events—N (%)	41 (20.2)	28 (13.9)	0.090*
Total number of adverse events—N	52	29	0.019**
Number of ≥ grade 3 adverse events—N	1	1	
Number of serious adverse events—N	0	0	
**Drug associated adverse events**^**a**^			
Number of participants reporting events—N (%)	10(4.9)	9(4.5)	0.820*
Total number of adverse events—N	15	9	0.320**
Allergic reaction or bronchospasm	2	0	
Fatigue or insomnia	4	4	
Gastrointestinal symptoms	2	1	
Respiratory symptoms	2	0	
Neuropathy or taste alteration	3	3	
Palpitation	1	0	
Rash	1	1	

Adverse events were rated by the principal investigator according to National Cancer Institute, Common Terminology Criteria for Adverse Events, version 3.0.

^a^Certainly or likely related to the study drug as rated by the principal investigator.

*p value calculated using the chi-squared test.

**p value calculated using repeated measures ANOVA.

## Discussion

In repeat blood donors with low iron stores, we found that intravenous supplementation with 800 mg ferric carboxymaltose did not result in a clinically relevant decrease in self-perceived fatigue compared to placebo. Our trial also revealed no significant differences in physical, emotional, and mental fatigue, general psychological distress, sleep quality, or general current health status between the iron and the placebo groups. Subgroup analyses of gender, participants with ferritin values < 25 µg/L, < 20 µg/L, < 15 µg/L and < 10 µg/L and baseline fatigue of ≥ 4 points on the NRS were consistent with our main findings of no significant difference between iron and placebo infusion groups.

Our findings align with an earlier, smaller study of oral iron replacement in iron-depleted blood donors^32^, with a recent intravenous iron study in female first time donors, in which fatigue was measured as secondary outcomes^34^, and with a large cohort study with more than 16,000 blood donors investigating the relationship between iron deficiency and self-reported mental or physical health^35^. Together, the evidence from these studies does not support the idea that NAID is a generalizable health problem in blood donors. In contrast, intravenous iron slightly increased hemoglobin and normalized ferritin in the iron supplementation group, while both parameters were unchanged in the placebo group. While these findings are compatible with the presence of a functional iron deficiency in erythropoietic cells, the absence of an improvement in participant reported outcomes of fatigue and general well-being suggests that functional iron deficiency in other tissues may not be a clinically relevant concept. We observed a rather low mean hemoglobin level in the lower range of normal in the study participants at baseline (Table 1). Although we enrolled the donors 4–6 weeks after the last blood donation, an ongoing decrease of the hemoglobin level by the donation is still possible. The increase of hemoglobin levels in the participants receiving iron supplementation suggests that individualised target hemoglobin levels for some repeat blood donors may be more appropriate.

Our findings vary compared to studies in premenopausal non-donor women that found a decrease of fatigue after iron supplementation^20,36–38^. In this specific population, studies with oral iron supplementation found a treatment effect on fatigue even in participants with very moderate iron deficiency of serum ferritin levels up to 50 µg/L^21,36^. With intravenous iron, an effect was only demonstrated when the deficiency was severe (ferritin < 15 µg/L)^37,38^. The difference may indicate a reporting bias among participants who deduced their treatment allocation, demonstrating the impact of incomplete blinding due to the treatment-related symptoms in trials using oral iron supplementation.

Our findings need to be discussed in the context of the INTERVAL parallel group, pragmatic, randomized trial which had included over 45,000 male and female whole blood donors across England^14^. In that trial, a reduction in inter-donation intervals over 2 years resulted in a modest increase in the prevalence of symptoms potentially related to blood donation, including self-reported tiredness. Although participants feeling more tired than usual had lower ferritin levels at 2-year examination, decrease in serum ferritin and hemoglobin concentrations explained only a small part of the symptoms linked with increased frequency donation. There could also be alterations in metabolic, immune and neurological pathways due to the shortening of donation intervals contributing to fatigue which are not directly related to the ferritin concentration. The role of the brain as an active organ for homeostatic control in the perception of bodily symptoms like fatigue is increasingly acknowledged^39^. In this understanding, a sustained deviation of autonomic, neuroendocrine and immunological states will prompt the brain to generate for instance fatigue, motivating a person to exercise adaptive behaviors in order to alleviate fatigue^40^.

### Strength and limitations

Our study was well blinded for both participants and study personnel and mitigated reporting bias, overcoming shortcomings in patient blinding due to gastrointestinal symptoms, including change of stool colour, in previous trials using oral iron supplementation^21,32,36,41^, or from the lack of investigator blinding in another trial of intravenous iron^38^. The loss of information due to attrition was very low, highlighting the quality of our data. In contrast to an earlier study in blood donors (32) and studies in premenopausal women with fatigue^21,36–38^, we included an almost equal number of male iron-depleted participants, helping to fill in this data and information gap. A limitation of our design may be the relatively high ferritin level (50 µg/L) for study inclusion, chosen on the basis of other studies on iron supplementation^36,37^. However, the pre-specified subgroup analysis of the primary outcome in donors with ferritin levels < 25 µg/L did not show significant interactions with treatment (Fig. 3). Furthermore, larger changes in self-reported fatigue should have been detectable, if present, due to the high number of study participants, the low mean ferritin level of the study population at baseline and the high number of participants with ferritin < 15 µg/L. However, we cannot rule out the possibility that blood donors with very high fatigue scores may benefit from iron supplementation. Nonetheless, there was no significant decrease in fatigue with iron supplementation in the subgroup of blood donors with a fatigue score ≥ 4 (n = 232; 57.3%), although statistical power was limited for this analysis. We assessed treatment response 6–8 weeks after iron or placebo infusion, but it may take several weeks longer for the fatigue-decreasing effect of iron supplementation to become fully apparent, as was suggested by a recent uncontrolled open-label study^42^. We did not investigate other outcomes, including cognition, restless legs syndrome and mood; for the latter two, sleep quality and general psychological distress are only proxy measures. Whether our findings are also applicable to non-anemic, non-donor premenopausal women with decreased ferritin levels would need to be explored in a subsequent randomized trial, as this intervention is widely used.

### Conclusions and implications for practice

Intravenous iron supplementation with iron carboxymaltose in repeat blood donors with iron deficiency without anemia had no effect on self-reported fatigue or general well-being, compared with placebo, although it was well tolerated in most participants. Our findings are not compatible with the notion of a NAID syndrome as a general problem in otherwise healthy, asymptomatic repeat blood donors. Besides short-lived effects immediately after phlebotomy, depleted iron stores of repeat blood donors were not found to be associated with self-rated fatigue or impaired general health. Our results do not support practices of regular ferritin monitoring or prophylactic iron supplementation in repeat blood donors without anemia. Even when ferritin levels are demonstrated to be low, iron supplementation cannot be recommended to improve self-reported fatigue or low general well-being in repeat blood donors.

## Methods

### Study design

ISUB was an investigator-initiated randomized single-centre placebo-controlled, superiority trial in healthy blood donors with depleted iron stores (ferritin ≤ 50 µg/L), comparing intravenous iron supplementation versus placebo. The trial was conducted in accordance with the Declaration of Helsinki and approved by the research ethics committee of the Canton of Bern (#010/11) and the Swiss Agency for Therapeutic Products, Swissmedic (2011DR3145). The study was registered with ClinicalTrials.gov, Number NCT01519830 on 01/27/2012. The CONSORT Checklist with information reported in this trial can be found as Supplementary Table 1S.

### Study participants

Blood donors who visited the blood donation centre of the Interregional Blood Transfusion Service of the Swiss Red Cross in Bern, Switzerland, were screened for iron deficiency. Capillary blood was collected to assess hemoglobin, and venous blood to measure serum ferritin. We enrolled men and women blood donors aged between 18 and 70 years, with a serum ferritin ≤ 50 μg/L, for whom this was not their first blood donation. Excluded were donors with anemia (hemoglobin level < 121 g/L for women; < 135 g/L for men), history of anaphylaxis, acute systemic infection of any kind, iron overload, intolerance of intravenous iron supplements, evidence of acute or chronic bleeding (especially gastrointestinal), disease or medication causing fatigue (judgement of the study physician), disease or medication not compatible with intravenous iron supplementation (judgement of the study physician), underweight (< 50 kg), overweight (> 85 kg for women, > 100 kg for men), pregnancy or breast feeding, and those unable to understand the questionnaire or to give informed consent. All participants provided written informed consent for both screening and the trial.

### Randomization and masking

Participants were centrally randomized 1:1 to receive intravenous iron or placebo using a locked, concealed web-based system (WebSpirit, 2mt Software GmbH, Ulm, Germany). Randomization was computer-generated, stratified by ferritin level (≤ 25 μg/L versus > 25 μg/L), gender, and fatigue [< 4 points versus ≥ 4 points on a 10-point numeric rating scale (NRS)]. Trial treatment assignment was double-blinded, so that neither study physicians and nurses involved in donor care and outcome assessment, nor participants were aware of treatment assigned. Intervention infusions were prepared in a separate building and administered by an independent team of study nurses, who provided no other care for study participants. To prevent unblinding by the colour of the trial infusion, infusion bags and injection sites were covered and nontransparent tubing was used to administer the study drug. The remaining study personnel performing data entry, queries and data management, and the statistician were all blinded to the allocated intervention until all primary and secondary analyses were completed.

### Intervention

Four to six weeks after the initial eligibility assessment and last blood donation, 800 mg ferric carboxymaltose (Ferinject) in 200 ml 0.9% sodium chloride solution or placebo (200 ml 0.9% sodium chloride solution) were administered intravenously over a period of 15 min. The interval of 4–6 weeks between the last blood donation/enrollment and iron supplementation was chosen to allow adequate red blood cell replacement and taking into account the fact that with a longer time interval, the ferritin level at screening could be increasingly influenced by various factors, including dietary changes. In accordance with clinical recommendations, the dose of 800 mg intravenous iron was chosen to guarantee adequate iron supplementation in heavy males, while posing no danger of iron overload in even the lightest study participants. To prevent distortion of study end points, additional blood donation was not allowed until outcome assessment was conducted 6–8 weeks later.

### Procedures

Eligible donors were invited by phone to participate in the trial and received information about the trial by mail. At a first visit 4–6 weeks after the last blood donation informed consent was obtained and a new blood sample analysed. Pregnancy was excluded in women of child-bearing age with a urine-based pregnancy test. The participant completed the psychometric questionnaire and received the infusion of the study drug. A second visit to assess treatment response was scheduled 6–8 weeks after infusion. At this visit, we also repeated blood sampling and completion of the psychometric questionnaire and assessed adverse events.

Laboratory analyses were performed at the first visit before treatment and at the second visit. One 2.7 ml tube containing EDTA was drawn for full blood count (Advia 2120i, Siemens Healthcare, Zürich, Switzerland). The serum resulting from a 10 mL blood tube without anticoagulant was frozen in aliquots at − 70 °C and used for later batch analysis of serum ferritin (FERR4 Cobas on a Hitachi 912 analyzer, Roche Diagnostics GmbH, Mannheim, Germany).

### Outcomes

The pre-specified primary outcome was self-rated average fatigue during the past 7 days, assessed 6–8 weeks after randomization and infusion of either iron or placebo, using a numeric rating scale (NRS) from 1 (no fatigue at all) to 10 (extreme fatigue)^21^. Secondary outcomes were self-perceived change in fatigue between baseline and 6–8 weeks after randomization, assessed on a NRS between − 10 and + 10; general well-being 6–8 weeks after randomization, measured by several validated self-rating questionnaires (30-item Multidimensional Fatigue Symptom Inventory-Short-Form (MFSI-SF)^43,44^, 27-item Symptom Checklist (SCL-27)^45^, 4-item Jenkins Sleep Questionnaire (JSQ)^46,47^, EuroQol-5D (EQ-5D)^48^); and blood parameters (hemoglobin and ferritin levels) 6–8 weeks after randomization. Safety outcomes were any adverse events, serious adverse events, and drug-associated adverse events reported by the participants after study drug infusion at the first visit and during outcome assessment at the second visit. These were rated by the principal investigator (PK) according to National Cancer Institute, Common Terminology Criteria for Adverse Events, version 3.0^49^. The study nurse and principal investigator were masked to the assigned treatment.

### Statistical analysis

A sample size of 163 participants per group yielded > 95% power to detect a clinically relevant difference of 1.0 on the 10-point fatigue NRS ranging from 1 to 10 at a two-sided alpha of 0.05, assuming a typical standard deviation of 2.5. The number of participants per trial arm was increased to 200, to enable us to detect a statistical interaction in subgroups with approximately 85% power, assuming a difference in the fatigue rating score of > 1.5 points. The protocol pre-specified the use of analyses of covariance for all continuous outcomes, adjusted for the outcome’s baseline values.

We performed pre-specified subgroup analyses and interaction tests according to ferritin at baseline (≤ 25 μg/L vs.  > 25 μg/L), gender, and fatigue at baseline (< 4 vs.  ≥ 4 points on the NRS). Analyses were based on the intention-to-treat principle, including all randomized patients according to their allocation^50^, using multiple imputation to impute missing outcome data^51^. We conducted a sensitivity analysis based on the per-protocol patient population. P-values and 95% confidence intervals (CIs) are two-sided. Analyses were performed in Stata version 12 software (StataCorp, College Station, Texas, USA) by an independent statistician of an academic clinical trials unit (CTU Bern, Switzerland) who was unaware of group assignment. Data were interpreted and conclusions formulated prior to unblinding investigators.

## Supplementary information

Supplementary Information 1.

Supplementary Information 2.

Supplementary Information 3.

## Data Availability

Deidentified individual participant data and the data analysis plan are available from the corresponding author on reasonable request.
