# Supplementary material for: The effects of intravenous iron supplementation on fatigue and general health in non-anemic blood donors with iron deficiency: a randomized placebo-controlled superiority trial
Source: Sci Rep. 2020 Aug 26;10:14219. doi: 10.1038/s41598-020-71048-0 (PMC7449957; doi:10.1038/s41598-020-71048-0)
Supplement: Supplementary file 2 — Supplementary Information 2. [file 41598_2020_71048_MOESM2_ESM.doc]

Bedeutung des Eisenmangels für das Blutspendewesen:

Auswirkung einer Eisensubstitution auf Müdigkeit und allgemeines Wohlbefinden bei gesunden Blutspendern

(ISUB : Iron substitution in blood donors)

**Version #1.2 vom 8.8.2011**

**Principal investigator: Dr. med. Peter Keller**

Universitätsklinik für Hämatologie und Hämatologisches

Zentrallabor, Inselspital / Universitätsspital, 3010 Bern

Datum: Unterschrift:

…………………………………….…. ……………………………………………………….

**Co-investigator: Dr. med. Stefano Fontana**

Blutspendedienst SRK Bern AG, 3008 Bern

**Sponsor: Universitätsklinik für Hämatologie**

Dr. P. Keller, Inselspital Bern

**Monitor : Clinical trials Unit**

Prof. P. Jüni, Inselspital Bern

Weitere Mitarbeiter und involvierte Institutionen:

- Prof. Dr. med. P. Jüni

Institut für Sozial und Präventivmedizin, Universität Bern

- Prof. Dr. med. R. von Känel

Chefarzt Psychosomatische Medizin, Inselspital Bern

- Prof. Dr. med. B. Lämmle

Direktor, Universitätsklinik für Hämatologie, Inselspital Bern

- Dr. Ch. Niederhauser, Blutspendedienst SRK Bern AG, 3008 Bern
- Blutspendezentrum Bern mit Mitarbeitern

Murtenstrasse 42, 3008 Bern, Blutspendedienst SRK Bern AG

- Clinical Trials Unit mit Mitarbeitern, Leitung Prof. P. Jüni, Inselspital Bern
- Clinical Investigation Unit mit Mitarbeitern

Leitung Prof. H.P. Marti, Inselspital Bern

# Inhaltsverzeichnis

[1. Zusammenfassung 4](#__RefHeading___Toc284008208)

[2. Hintergrundinformationen und aktueller Wissensstand 5](#__RefHeading___Toc284008209)

[2.1. Eisen: Physiologische Aspekte 5](#__RefHeading___Toc284008210)

[2.2. Eisenmangel ohne Anämie 5](#__RefHeading___Toc284008211)

[2.3. Eisenmangel im Bluspendewesen 6](#__RefHeading___Toc284008212)

[2.4. Studienmedikation 7](#__RefHeading___Toc284008213)

[3. Zielsetzungen und Zweck 8](#__RefHeading___Toc284008214)

[3.1. Grundlagen zum Studienkonzept 8](#__RefHeading___Toc284008215)

[3.2. Studienziele 8](#__RefHeading___Toc284008216)

[3.3. Fragestellung 9](#__RefHeading___Toc284008217)

[3.3.1. Übersichtsstudie 9](#__RefHeading___Toc284008218)

[3.3.2. Hauptfrage der Interventionsstudie 9](#__RefHeading___Toc284008219)

[3.3.3. Sekundäre Studienfragen: 9](#__RefHeading___Toc284008220)

[3.4. Hypothese 9](#__RefHeading___Toc284008221)

[4. Studiendesign 10](#__RefHeading___Toc284008222)

[4.1. Überblick 10](#__RefHeading___Toc284008223)

[4.2. Studienschema 10](#__RefHeading___Toc284008224)

[4.3. Endpunkte 11](#__RefHeading___Toc284008225)

[4.3.1. Übersichtsstudie 11](#__RefHeading___Toc284008226)

[4.3.2. Interventionsstudie 11](#__RefHeading___Toc284008227)

[4.4. Ablauf Übersichtsstudie 11](#__RefHeading___Toc284008228)

[4.5. Ablauf der Interventionsstudie 12](#__RefHeading___Toc284008229)

[4.5.1. Studieneinschluss / erste Studienvisite 12](#__RefHeading___Toc284008230)

[4.5.2. Zweite Studienvisite / Studienabschluss 12](#__RefHeading___Toc284008231)

[4.5.3. Studienmedikation 13](#__RefHeading___Toc284008232)

[4.6. Randomisierung und Verblindung in der Interventionsstudie 13](#__RefHeading___Toc284008233)

[4.7. Vorzeitiger Studienaustritt 13](#__RefHeading___Toc284008234)

[4.8. Probandenbetreuung 14](#__RefHeading___Toc284008235)

[5. Auswahl der Versuchspersonen 15](#__RefHeading___Toc284008236)

[5.1. Übersichtsstudie 15](#__RefHeading___Toc284008237)

[5.1.1. Einschlusskriterien: 15](#__RefHeading___Toc284008238)

[5.1.2. Ausschlusskriterien: 15](#__RefHeading___Toc284008239)

[5.2. Interventionsstudie 15](#__RefHeading___Toc284008240)

[5.2.1. Einschlusskriterien: 15](#__RefHeading___Toc284008241)

[5.2.2. Ausschlusskriterien: 15](#__RefHeading___Toc284008242)

[6. Messparameter 16](#__RefHeading___Toc284008243)

[6.1. Übersichtsstudie 16](#__RefHeading___Toc284008244)

[6.1.1. Fragebogen 16](#__RefHeading___Toc284008245)

[6.1.2. Laboranalysen 16](#__RefHeading___Toc284008246)

[6.2. Interventionsstudie 16](#__RefHeading___Toc284008247)

[6.2.1. Fragebogen 16](#__RefHeading___Toc284008248)

[6.2.2. Laboranalysen 17](#__RefHeading___Toc284008249)

[7. Sicherheit 18](#__RefHeading___Toc284008250)

[7.1. Sicherheitsprofil des Studienmedikaments 18](#__RefHeading___Toc284008251)

[7.2. Definitionen 18](#__RefHeading___Toc284008252)

[7.2.1. Adverse event (AE) 18](#__RefHeading___Toc284008253)

[7.2.2. Serious adverse event (SAE) 18](#__RefHeading___Toc284008254)

[7.2.3. Adverse drug reaction (ADR) 18](#__RefHeading___Toc284008255)

[7.2.4. Suspected unexpected serious adverse reactions (SUSAR) 18](#__RefHeading___Toc284008256)

[7.3. Dokumentation und Beurteilung von Nebenwirkungen 19](#__RefHeading___Toc284008257)

[7.4. Notifikation von Nebenwirkungen und jährlicher Sicherheitsbericht 19](#__RefHeading___Toc284008258)

[7.5. Schwangerschaft 19](#__RefHeading___Toc284008259)

[8. Poweranalyse und Statistik 20](#__RefHeading___Toc284008260)

[9. Studienspezifische Vorsichtsmassnahmen 21](#__RefHeading___Toc284008261)

[9.1. Schwangerschaft 21](#__RefHeading___Toc284008262)

[10. Pflichten des Prüfers 22](#__RefHeading___Toc284008263)

[10.1. Einhaltung der gesetzlichen Bestimmungen 22](#__RefHeading___Toc284008264)

[10.2. Berichterstattung 22](#__RefHeading___Toc284008265)

[10.3. Versicherung 22](#__RefHeading___Toc284008266)

[11. Ethische Überlegungen 23](#__RefHeading___Toc284008267)

[11.1. Übersichtsstudie 23](#__RefHeading___Toc284008268)

[11.2. Interventionsstudie 23](#__RefHeading___Toc284008269)

[12. Qualitätssicherung 24](#__RefHeading___Toc284008270)

[12.1. Monitoring 24](#__RefHeading___Toc284008271)

[12.2. Inspektionen 24](#__RefHeading___Toc284008272)

[12.3. Behandlung von Daten 24](#__RefHeading___Toc284008273)

[12.3.1. Case Report Forms 24](#__RefHeading___Toc284008274)

[12.3.2. Archivierung 24](#__RefHeading___Toc284008275)

[12.3.3. Elektronische Datenbank 25](#__RefHeading___Toc284008276)

[12.3.4. Datenvalidierung 25](#__RefHeading___Toc284008277)

[12.3.5. Datenanalyse und Archivierung 25](#__RefHeading___Toc284008278)

[12.4. Umgang mit Blutproben 25](#__RefHeading___Toc284008279)

[13. Zusatzinformationen für die Notifizierung (Swissmedic) 26](#__RefHeading___Toc284008280)

[13.1. Drug Accountability 26](#__RefHeading___Toc284008281)

[13.2. Erfassung der Compliance 26](#__RefHeading___Toc284008282)

[13.3. Beschriftung des Prüfpräparates 26](#__RefHeading___Toc284008283)

[13.4. Publikation der Resultate 27](#__RefHeading___Toc284008284)

[14. Literatur 28](#__RefHeading___Toc284008285)

1. Zusammenfassung

**Hintergrund:**

Tiefe Eisenspeicher bei Blutspendern sind ein häufiges Phänomen. Ob ein Eisenmangel aber Auswirkungen auf die Gesundheit der Spender hat, ist ungenügend studiert. Die Grenz­werte für einen klinisch relevanten Eisenmangel sind sowohl in der allgemeinen Medizin wie speziell bei Blutspendern sehr umstritten. Einige schlecht kontrollierte Studien mit oraler Eisensubstitution bei regelmässigen Blutspendern konnten zeigen, dass die langfristige Fähigkeit und Bereitschaft zum Blutspenden dadurch verbessert werden konnte. Die Studien waren aber nicht in der Lage, einen positiven Effekt auf die Gesundheit der Spender nachzuweisen. Bis heute ist es deshalb umstritten, ob bei regelmässigen Blutspendern ein Eisen­mangel gesucht und substituiert werden soll.

**Ziel des Projekts:**

- Detaillierte Beschreibung der Eisenmangelproblematik bei einer grossen Gruppe von Blutspendern.
- Klärung des Zusammenhangs zwischen tiefem Speichereisen und potentiell damit ver­bundenen Beschwerden, insbesondere Müdigkeit.
- Definition einer unteren Eisenspeichergrenze bei Blutspendern, ab welcher somatische und psychische Beschwerden auftreten.
- Nachweis der Effektivität einer intravenösen Eisensubstitution und dadurch formaler Beweis des Zusammenhangs zwischen Eisenmangel und Symptomen.
- Nachweis der Sicherheit einer intravenösen Eisensubstitution bei Blutspendern.

**Studienaufbau:**

- Deskriptive Übersichtsstudie bei grossem Blutspenderkollektiv zur Erfassung des Eisen­status und der damit verbundenen Beschwerden
- Doppelblinde, randomisierte, placebokontrollierte Interventionsstudie mit intravenöser Verabreichung von Eisencarboxymaltose (Ferinject®) oder Placebo bei gesunden Blutspendern mit vermindertem bis tiefnormalem Speichereisen

**Primärer Endpunkt der Interventionsstudie:**

Verbesserung der subjektiv empfundenen Müdigkeit nach intravenöser Eisensubstitution im randomisierten, doppelblinden Vergleich mit Placebo.

**Wichtigste Einschlusskriterien der Interventionsstudie:**

Blutspendetauglichkeit, Serumferritin ≤50.0 μg/l

**Wichtigste Ausschlusskriterien der Interventionsstudie:**

Anämie, Unverträglichkeit intravenösen Eisens, durchgemachte anaphylaktische Reaktion, aktive Krankheiten, bekannte Eisenüberladung, akute oder chronische Blutung, Körperge­wicht <50 kg und >85 kg (Frauen) / >100 kg (Männer), Alter <18 oder >65 Jahre

1. **Hintergrundinformationen** und aktueller Wissensstand
   1. Eisen: Physiologische Aspekte

Der menschliche Körper enthält je nach Geschlecht und Grösse ungefähr 3 bis 4.5 Gramm Eisen. Der grösste Teil davon befindet sich als Häm-gebundenes Eisen in den Erythro­zyten^1^. Ein Gramm Hämoglobin enthält 3.4 mg Eisen. Daraus wird ersichtlich, dass durch eine Vollblutspende von 450 ml dem Körper zwischen 180 und 250 mg Eisen entzogen wird. Neben seiner lebensnotwendigen Rolle als Sauerstoffträger im roten Blutfarbstoff ist Eisen noch in vielen anderen Proteinen und Enzymen für zahlreiche es­sentielle Funktio-nen verantwortlich. Grundlage dieser wichtigen biologischen Rolle ist das hohe Redox-Potential des Eisens. Auf demselben Mechanismus basieren aber auch die potentiell toxischen Eigenschaften von überschüssigem Eisen im Körper, indem es über eine vermehrte Radikalbildung zur Schädigung verschiedener Organe kommen kann. Die Eisenverteilung im Organismus ist deshalb gut reguliert. Kurzfristig wird überschüssiges Eisen von Makrophagen des retikuloendothelialen Systems aufgenommen. Langfristig wird Eisen in den Zellen hauptsächlich als Makrokomplex mit dem Eiweiss Ferritin gespeichert. Dement­sprechend widerspiegelt Serumferritin das Speichereisen des Körpers und dient in der Medizin als wichtigster Laborparameter zur Diagnose eines Eisenmangels.

- 1. Eisenmangel ohne Anämie

Das Vollbild eines schweren Eisenmangels ist unverkennbar mit allen Auswirkungen der Blutarmut wie Blässe, Kraftlosigkeit, Erschöpfung, Müdigkeit und vielen anderen Symptomen. Viel schwieriger einzuordnen sind leichtere Eisenmangelzustände, die noch nicht zu einer Blutarmut geführt haben. Dieses Thema ist in der Inneren Medizin nach wie vor stark umstrittene. Viele verschiedene Symptome wie zum Beispiel chronische Müdigkeit, Konzentrationsstörungen, Erschöpfungszustände, Depressionen oder Antriebsarmut werden dieser Konstellation zugeschrieben. Da Eisen in vielen essentiellen Enzymen, zum Beispiel in den Muskeln und im Hirn eine zentrale Rolle spielt, sind solche Beschwerden theoretisch gut erklärbar.

Klinische Studien geben gewisse Hinweise, dass Eisenmangel ohne Anämie tatsächlich zu Beschwerden führt. Zum Beispiel konnte nach sechswöchiger peroraler Eisensubstitution bei nicht-anämen Frauen mit labormässigem Eisenmangel unter Ausdauertraining ein signifikant höherer Anstieg der aeroben Kapazität nachgewiesen werden^2^. Bei adoleszenten, US-amerikanischen Frauen mit Eisenmangel ohne Anämie führte eine achtwöchige perorale Eisensubstitution im Vergleich zur Placebo-Kontrollgruppe zu einer signifikanten Verbesserung verschiedener kognitiver Funktionen^3^. Anreicherung der Nahrung mit Eisen führte in einer anderen Untersuchung zu einer generellen Verbesserung verschiedener Gesundheitsmerkmale^4^. In einer wichtigen Studie aus dem Universitätsspital Lausanne wurden Frauen mit latentem Eisenmangel, die unter unerklärter Müdigkeit litten, placebokontrolliert mit peroralem Eisen behandelt. Mit Hilfe einer numerischen Messskala konnte eine signifikante Verbesserung der Müdigkeit gezeigt werden^5^. Diese Studie ist aber umstritten, da wegen der peroralen Eisensubstitution mit Schwarzverfärbung des Stuhls und gastrointestinalen Beschwerden das Verum und das Placebo nicht wirklich blind verabreicht werden konnten und ein Placeboeffekt daher nicht sicher ausgeschlossen werden konnte.

Durch Eisenmangel ohne Anämie ausgelöste Beschwerden sind generell mild und unspe-zifisch. Sie können einfach mit Beschwerden anderer Krankheiten oder alltäglichen Unpässlichkeiten verwechselt werden. Oft ist es für den Arzt schwierig zu entscheiden, ob ein postulierter Eisenmangel tatsächlich für die Beschwerden des Patienten verantwortlich ist. Der Zustand eines Eisenmangels ohne Anämie wurde in den letzten Jahren vor allem durch die Pharmaindustrie als eigenständiges Krankheitsbild propagiert. Auch selbsternannte Eisenexperten in spezialisierten Arztpraxen („Eisenzentren“) griffen das Thema auf und praktizieren grosszügige, intravenöse Eisensubstitutionen. Durch Berichte in der Laien­presse wurde dadurch in weiten Bevölkerungskreisen eine beträchtliche Unruhe ausgelöst^6^.

Die wissenschaftlichen Grundlagen zur grosszügigen Eisensubstitution bei Eisenmangel ohne Anämie sind ungenügend. Durch Anpreisung hoher Eisengrenzwerte und häufigen Eiseninfusionen sind negative Gesundheitsfolgen bei einer grossen Zahl von Menschen nicht auszuschliessen. Aufgrund der heutigen Datenlage herrscht Unklarheit, oberhalb welchem Grenzwert der Eisenparameter ein klinisch relevanter Eisenmangel ausge-schlossen werden kann und keine Eisensubstitution indiziert ist.

- 1. Eisenmangel im Bluspendewesen

Bei Blutspendern sind tiefe Eisenspeicher ein bekanntes Phänomen^7^. Dass wiederholte Spenden einen Eisenmangel auslösen oder verstärken, ist allgemein akzeptiert^8^. Eine gepoolte Analyse^7^ von vier Studien^9-12^ fand ein durchschnittliches Ferritin von 124 μg/l bei männlichen Erstspendern und ein Ferritin von 47 μg/l bei männlichen Mehrfachspendern. Bei Frauen betrugen die entsprechenden Ferritinwerte 46 μg/l und 25 μg/l. Die Entwicklung eines Eisenmangels scheint dabei nicht so sehr von der Gesamtzahl der Spenden, sondern von deren Häufigkeit pro Zeit abzuhängen^10,13^. Daten vom Zürcher Blutspendedienst SRK bei langjährigen Blutspendern zeigten auch, dass sich das Eisen-gleichgewicht bei sehr häufigem Spenden langfristig stabilisiert^14^. Dass der Organismus in der Lage ist, bei häufigem Blutspenden die Eisenaufnahme aus dem Darm massiv zu steigern, beweist eine Studie mit sogenannten Superspendern (13 Spenden pro 2 Jahre)^15^. Hepcidin, welches die enterale Eisenresorption verhindert, war bei diesen Probanden deutlich vermindert. Gleiche Resultate werden auch aus dem Zürcher Blutspendedienst SRK berichtet^16^.

Die meisten Daten zum Eisenstatus bei Blutspendern stammen aus kleinen, schlecht kontrollierten Studien und sind zum Teil widersprüchlich. In einer frühen Studie aus Albuquerque, NM, mit 1021 unselektionierten Blutspendern fand sich bei 23.4% der Frauen und 7.7% der Männer ein Serumferritin von kleiner 12 μg/l^10^. Eine norwegische Studie ergab bereits bei 21.7% der weiblichen und 4.6% der männlichen Neuspender eine Anämie (Hämoglobin unter der Spendefähigkeitsgrenze)^17^. Bei 56.9% dieser Frauen war das Ferritin <30 μg/l und bei 20% <15 μg/l. Nur bei 3.3% dieser Männern war das Ferritin <30 μg/l und nur bei einem unter 15 μg/l^17^. Für die Schweiz existieren als Poster publizierte Daten aus dem Zürcher Blutspendedienst SRK^18^: Bei 1539 Spendern jünger als 40 Jahre fand sich in 25.9% ein Ferritin kleiner 15 μg/l. Dies war der Fall bei 51.7% der Frauen und bei 4.7% der Männer. Interessanterweise war auch bei 25.1% gesunder Neuspender das Ferritin kleiner 15 μg/l.

Auch im Bereich des Blutspendewesens ist heute ungeklärt, ob eine Eisendepletion tatsächlich gesundheitliche Auswirkungen hat. Zwar kann nachgewiesen werden, dass mittels peroraler Eisensubstitution das Gesamtkörpereisen bei häufig blutspendenden Männern konstant gehalten und bei Frauen sogar langsam erhöht werden kann^19^. Auch wurde gezeigt, dass durch perorale Eisensubstitution die Spendefähigkeit verbessert und eine Spenderverlust vermieden werden kann^20,21^. Es existiert aber kein eindeutiger Beweis, dass ein Eisenmangel bei Blutspendern wirklich mit Beschwerden korreliert und eine Eisensubstitution Beschwerden oder gesundheitliche Schäden vermindern kann. Schliesslich ist unklar, ab welchem unteren Grenzwert des Ferritins ein Eisenmangel symptomatisch wird und behandelt werden muss. Sollte tatsächlich bewiesen werden können, dass sich leere Eisenspeicher negativ auf die Gesundheit der Spender auswirken, hätte dies direkte Folgen für die Blutspendepraxis. Eine regelmässige Kontrolle der Eisenspeicher müsste als obligatorisch erklärt werden und allenfalls längere Spendeintervalle oder eine regelmässige Eisensubstitution eingeführt werden.

- 1. Studienmedikation

Eisencarboxymaltose (Ferinject®) ist in der Schweiz seit 2008 für die intravenöse Eisensubstitution durch Swiss­medic zugelassen und krankenkassenpflichtig. Die Indikation sind labormässig nachgewiesene Eisenmangelzustände, die auf orale Eisensubstitution nicht ansprechen oder bei welchen eine orale Substitution aus medizinischen Gründen kontraindiziert ist. In die vorliegende Studie werden nur Probanden eingeschlossen, bei welchen mit einem Ferritin ≤50 μg/l ein Eisenmangel sehr wahrscheinlich ist. Wie unter Kapitel 3.1 diskutiert, muss aus studientechnischen Gründen die parenterale Verabreich-ung der oralen Eisensubstitution vorgezogen werden. Die Anwendung von Ferinject® dient dem pathophysiologischen Studium des noch nicht vollständig verstandenen Phänomens eines Eisenmangels ohne Anämie. Bezüglich Ansprechen werden deshalb nur die studienspezifischen Endpunkte erfasst. Zudem soll das Nebenwirkungsspektrum von Ferinject® bei gesunden Blutspendern beschrieben werden.

Mit der Eisencarboxymaltose-Infusion sollen die Eisenspeicher aufgefüllt werden. Ein zusätzliches Eisendefizit durch Hämoglobin-gebundenes Eisen besteht bei unseren Probanden nicht. Grob geschätzt betragen die Eisenspeicher bei einer Frauen 500 mg und bei einem Mann 1000 mg Eisen. Für die Studie wurde eine Einheitsdosis von 800mg Eisencarboxymaltose gewählt. Diese Dosis garantiert bei beiden Geschlechtern sowie bei leichten und schweren Personen ein genügendes Füllen der Eisenspeicher. Gleichzeitig sollte bei der gewählten Dosis das Ferritin im Gleichgewicht bei keinem Probanden über die obere Labornorm ansteigen. Damit ist eine langfristige Toxizität durch Eisenüberladung nicht zu befürchten.

1. Zielsetzungen und Zweck
   1. Grundlagen zum Studienkonzept

Die klinischen Auswirkungen eines Eisenmangels ohne Anämie sind schwierig zu erfassen, da sie mit vielen anderen somatischen Krankheiten oder psychischen Beschwer-den überlappen. Wegen dem schleichenden Auftreten könnten sie lange unbe­merkt bleiben. Die Wahrnehmung eines Eisenmangels kann zudem je nach Persönlich­keitsstruktur des Patienten stark variieren. Bei der Gruppe der Blutspender, die im Ver­gleich zur Durchschnittsbevölkerung sehr motiviert und positiv eingestellt sind, könnte man erwar­ten, dass Beschwerden eher spät bemerkt werden.

Um die milden und unspezifischen Symptome eines Eisenmangels zu messen, ist eine sehr sensitive Methode erforderlich. Der Einfluss von interindividuellen und subjektiven Unterschieden ist möglichst zu minimieren. Die Erfassung von Eisenmangel-assoziierten Beschwerden alleine mit Hilfe eines Fragebogens würde darum kaum gelingen. Durch randomisierte und verblindete Verabreichung von Eisen oder Placebo und Messung der subjektiv wahrgenommenen Müdigkeit vor und nach Substitution des Eisendefizits können diese Probleme weitgehend umgangen werden. Die gute Sensitivität einer numerischen Messskala zur Quantifizierung des Müdigkeitsempfindens ist gut etabliert^5^.

Unser Studienkonzept ist in der Lage, zahlreiche Schwierigkeiten im Zusammenhang mit Eisenmangel-assoziierten Beschwerden zu vermeiden. Nur die Wahl einer intravenösen Eisensubstitution erlaubt eine verlässliche Verblindung, da perorale Eisenpräparate aufgrund ihrer gastrointestinalen Nebenwirkungen immer vom Probanden erkannt werden. Die intravenöse Verabreichung löst auch das Problem der schlechten Medikamenten-einnahme, das aufgrund der gastrointestinalen Nebenwirkungen bei peroralem Eisen unvermeidbar ist^19^. Die intravenöse Infusion führt zu einem raschen Auffüllen der Eisenspeicher. Eine Verbesserung des Befindens wird dadurch für den Probanden viel besser spürbar, was die Sensitivität der Untersuchung wesentlich erhöht. Beide Probleme dürften in einer ähnlichen, aktuell in Lausanne laufenden Studie^22^ mir peroralem Eisen eine wesentlich grössere Rolle spielen.

Der Aufbau unserer Studie erlaubt die bestmögliche Verblindung zwischen Verum- und Placebo-Medikation. Im schwierigen Symptomenkomplex der Eisenmangel-assoziierten Beschwerden ist dies absolut relevant.

- 1. Studienziele

In einer Übersichtsstudie bei allen Blutspendern am Blutspendezentrum Bern soll das Ausmass und die Häufigkeit des Eisenmangels bei Blutspendern sauber erfasst werden. Die subjektiv berichteten Beschwerden sollen mit den verschiedenen Eisenparametern und dem bisherigen Spendeverhalten der Probanden korreliert werden.

In einer Interventionsstudie mit intravenöser Eisensubstitution im doppelblinden Vergleich mit einem Placebopräparat soll untersucht werden, ob sich die subjektiv empfundene Müdigkeit und andere Allgemeinsymptome verbessern und dadurch gesundheitliche Auswirkungen eines Eisenmangels ohne Anämie bewiesen werden können.

- 1. Fragestellung
     1. Übersichtsstudie

Beschreibung der Häufigkeit und der Auswirkungen eines Eisenmangels bei einem grossen Kollektiv von Blutspendern.

- - 1. Hauptfrage der Interventionsstudie

Kann durch intravenöse Substitution von Eisen bei Blutspendern ohne Anämie eine Verbesserung der subjektiven Müdigkeitsempfindens nachgewiesen werden?

- - 1. Sekundäre Studienfragen:

Verspürt der Proband nach intravenöser Eisensubstitution subjektiv eine Veränderung seiner Müdigkeit?

Kann mit Hilfe verschiedener psychometrischer Tests eine Verbesserung des allgemeinen und psychischen Wohlbefindens nach intravenöser Eisensubstitution nachgewiesen werden?

Kann nach intravenöser Eisensubstitution ein Anstieg des Hämoglobins und eine Verbesserung des Eisenstatus nachgewiesen werden?

Wie wird eine intravenöse Eisensubstitution bei gesunden Blutspendern vertragen?

Kann bei folgenden Subpopulationen eine Verbesserung der subjektiv empfundenen Müdigkeit festgestellt werden: Frauen, Männern, Ferritin <25 μg/l, Ferritin >25 μg/l, vorbestehende Müdigkeit auf der numerischen Messskala <4 oder >4?

- 1. Hypothese

Auch ein leichter Eisenmangel, der noch nicht zu einer Blutarmut geführt hat, kann bei einem Teil der Menschen eine relevante Beeinträchtigung des Allgemeinbefindens verursachen. Die Beschwerden sind aber so mild und unspezifisch, dass sie nur durch Vergleich des Zustandes vor und nach Eisensubstitution und unter sorgfältiger Vermeidung eines subjektiven Bias bewiesen werden können.

1. Studiendesign
   1. Überblick

Das Projekt besteht aus zwei Teilen, einer Übersichtsstudie in einem Blutspenderkollektives und einer Interventionsstudie mit intravenöser Eisenverabreichung bei Spendern mit Ferritinwerten ≤50 μg/l.

Als Screening für die Interventionsstudie wird bei allen Blutspendern die einwilligen prospektiv während ca. 3 Monate das Ferritin gemessen und ein Fragebogen zur Spendeanamnese und zu Symptomen des Eisenmangels zum Ausfüllen abgegeben.

Der Hauptteil des Projekts ist eine randomisierte, doppelblinde, placebokontrol­lierte Studie mit intravenöser Verabreichung von Eisencarboxymaltose (Ferinject®) bei männlichen und weiblichen Blutspendern mit vermindertem oder tiefnormalem Speicherei­sen. Primärer Endpunkt ist eine Verbesserung der subjektiv empfundenen Müdigkeit.

- 1. **Studienschema**

**Erste**

**Blutspende**

**Einschluss**

**Interventionsstudie**

**Abschlussvisite**

**Zweite Blutspende**

6-8 Wochen (42-56 Tage)

4 bis 6 Wochen

**Übersichtsstudie Blutspenderkollektiv**

Hämoglobin kapillär; Blutentnahme: Ferritin, Weitere (siehe Text)

Fragebogen „Bedeutung des Eisenmangels bei Blutspendern“

**Studienende**

**Ausfüllen der Fragebogen**

Blutentnahme: Blutbild (Advia 120), Ferritin, Weitere (siehe Text, Reserveserum -70° C)

**Auswahl der Probanden (Ferritin ≤50 μg/l)**

Telephonische Einladung zur Studienteilnahme

**Unterschrift der Einverständniserklärung**

**Randomisierung** (mit Stratifizierung)

**Ausfüllen der Fragebogen**

Blutentnahme: Blutbild (Advia 120), Ferritin, Weitere (siehe Text, Reserveserum -70° C)

**Placebo**

Intravenöse Infusion über 15’

**Ferinject**

Intravenöse Infusion über 15’

- 1. Endpunkte
     1. Übersichtsstudie

Erfasst werden die Häufigkeit und der Schwere­grad des Eisenmangels sowie der mit Eisenmangel assoziierten subjekti­ven Beschwerden bei Blutspendern am Blutspendezentrum Bern. Zusätzlich soll der postulierte Zusammenhang zwischen Eisenman­gel, klinischen Symptomen und dem bisherigen Blutspendeverhalten untersucht werden. Zur Erfassung des Eisen­status werden mehrere Eisenparameter gemessen und die diagnostische Effizienz der verschiedenen Parameter verglichen.

- - 1. Interventionsstudie

**Primärer Endpunkt:**

- Unterschied im mittleren Müdigkeitsempfinden auf einer numerischen Messskala von 1 bis 10 zwischen der Ferinject- und der Placebo-Gruppe vier bis sechs Wochen nach Infusion des Studienpräparates

**Sekundäre Endpunkte:**

- Subjektiv wahrgenommene Änderung der Müdigkeit nach Verabreichung des Studienmedikaments, gemessen mit einer numerischen Messskala von -10 bis +10.

- Verbesserung des allgemeinen und psychischen Wohlbefindens nach intravenöser Eisensubstitution, gemessen mit mehreren psychometrischen Tests.

- Verlauf von Hämoglobin und Eisenparametern nach intravenöser Eisensubstitution.
- Verträglichkeit von intravenösem Eisen bei gesunden Blutspendern.
- Verbesserung der Müdigkeit bei folgenden Subpopulationen: Frauen, Männern, Ferritin <25, Ferritin >25, Müdigkeitskala <4, Müdigkeitskala >4 (siehe Stratifizierungskriterien)

Der Hauptendpunkt „Müdigkeit“ soll im umgangssprachlichen Sinne ohne weitere Definition erfragt werden. Deren Erfassung mittels einer numerischen Messskala, respektive einer visual-analogen Skala wurde in einer früheren Studie bei Frauen mit chronischer Müdigkeit und vermindertem Körpereisen bereits erfolgreich angewandt^5^. Mit Hilfe dieses Endpunkts war es möglich, eine klinisch relevante Reduktion der subjektiv empfundenen Müdigkeit statistisch signifikant nachzuweisen. Da „Müdigkeit“ von verschiedenen Menschen zum Teil sehr unterschiedlich erlebt wird, werden zusätzlich verschiedene Subqualitäten von „Müdigkeit“ als sekundärer Endpunkt mit Hilfe des Multi-dimensional fatigue symptom inventory, short form^23^ (MFSI-SF) erfasst.

- 1. Ablauf Übersichtsstudie

Während der Rekrutierungsphase für die Interventionsstudie (ca. 3 Monate) werden konsekutive alle Vollblutspender des Blutspendezentrums SRK Bern dazu eingeladen, an einer Studie zur Untersuchung Ihres Eisenstatus teilzunehmen. Dazu wird jedem Spender mit dem Spendefragebogen ein Informationsblatt und die Einverständnis­erklärung abgegeben. Gibt der Spender seine schriftliche Einwilligung, wird vor der Blutspende zusätzlich ein 2.7 ml EDTA-Röhrchen und ein 10 ml Serum­röhrchen abgenommen. Daraus wird das Serumferritin bestimmt. Bei einer Zufallsauswahl werden das Blutbild und weitere Eisenparameter bestimmt.

Der Proband wird gebeten einen Fragebogen auszufüllen, der Fragen zur Spende-vorgeschichte und zu Eisenmangel-assoziierte Beschwerden umfasst. Die Beantwortung beansprucht ca. 10 bis 15 Minuten. Aus den Spenderunterlagen werden durch die Studienassistentin Informationen zur medi­zinischen Vorgeschichte und aktuellen Medikation entnommen.

Falls der Proband nicht an der Interventionsstudie teilnimmt und das Ferritin sehr tief ist (≤50 μg/l), wird ihm das Resultat schriftlich mitgeteilt. Andernfalls kann er das Resultat telephonisch oder bei der nächsten Spende erfragen.

- 1. Ablauf der Interventionsstudie
     1. Studieneinschluss / erste Studienvisite

Für die Studie qualifizieren Spender mit einem Serumferritin ≤50 μg/l. Unter Berücksichtigung der Ein- und Ausschlusskriterien werden fortlaufend mögliche Probanden ausgewählt und telephonisch zur Studienteilnahme eingeladen. Bei vorläufi­gem Einverständnis wird die Probandeninformation zugeschickt und der Kandidat vier bis sechs Wochen nach seiner Blutspende für die erste Studienvisite aufgeboten.

Anlässlich der ersten Visite erfolgt die Information durch den Spendearzt. Bei defini­tiver Einwilligung zur Studienteilnahme mit unterschriebener Einverständniserklärung wird dem Probanden das Fragebogenpaket zum Ausfüllen abgegeben und eine Blutentnahme abgenommen. Daraus wird das Blutbild inklusive Erythrozytenindices (Advia 120), das Serumferritin sowie weitere Eisenparameter bestimmt. Da ein Teil der Analysen erst später stattfindet, wird Serum portioniert und bei -70° C eingefroren. Bei Frauen im gebärfähigen Alter wird ein Urin-Schwangerschaftstest abgenommen.

Sobald der Patient die Einwilligung unterschrieben hat, werden die Pflegefachpersonen der Clinical Investigation Unit informiert. Diese randomisieren und stratifizieren den Patienten mit Hilfe der Studiendatenbank und bereiten die Studienmedikation zu. Die fertige Infusion wird so verpackt, dass deren Farbe nicht erkannt werden kann. Die Infusion wird ins Blutspendezentrum gebracht und dort von der Pflegefachperson der CIU dem Patienten verabreicht. Um paravenöse Invasate mit Hautverfärbungen zu vermeiden, die auch die Verblindung beeinträchtigen könnten, wird das Studienmedikament ausschliesslich über eine Venenverweilkanüle (Venflon) infundiert.

- - 1. Zweite Studienvisite / Studienabschluss

Um den Effekt der Eisensubstitution nicht zu verwischen, darf ab der ersten Blutspende anlässlich des Screenings bis zur Abschlussvisite keine Vollblut-, Erythrozyten-, Thrombozyten- oder Plasmaspende erfolgen. Die zweite, abschliessende Visite erfolgt sechs bis acht Wochen nach Verabreichung des Studienpräparates (erlaubtes Zeitfenster 42 bis 56 Tage). Der Proband wird gebeten, das Fragebogenpaket ein zweites Mal auszufüllen. Zudem werden von der Studienkoordinatorin allfällige Nebenwirkungen auf die Studienmedikation erfragt. Anschliessend wird Blut entnommen und daraus nochmals das Blutbild und verschiedene Eisenparameter bestimmt. Wie­derum wird Material bei -70° C eingefroren. Die Studie ist nun für den Probanden abgeschlossen. Falls er dies wünscht, darf zum Schluss noch eine reguläre Blutspende durchgeführt werden.

Im Anschluss an diese Visite wird dem Probanden schriftlich mitgeteilt, ob er das Eisenpräparat oder das Placebo erhalten hat. Zudem werden ihm die Laborresultate der ersten Visite und der Ferritinwert der zweiten Visite zugeschickt.

- - 1. Studienmedikation

Eisencarboxymaltose (Ferinject®)

Eisencarboxymaltose ist in der Schweiz seit 2008 von Swissmedic zur Behandlung des Eisenmangels zugelassen. Das Medikament wird von der Firma Vifor Pharma AG gratis zur Verfügung gestellt. Die Verabreichung erfolgt in einer 0.9% NaCl-Trägerlösung als intravenöse Infusion. Die Dosis wurde folgendermassen festgelegt:

► **800mg Eisencarboxymaltose**

→ 16ml Ferinject in 200ml 0.9% NaCl als Kurzinfusion über 15 Minuten intravenös

Placebo

Als Placebo wird ungefärbte, 0.9% NaCl-Lösung verwendet. Ein mit dem Verum farbidentisches Placebopräparat ist nicht verfügbar. Um die Verblindung trotzdem zu gewährleisten, erfolgt die Verabreichung des Studienpräparates durch eine externe Pflegefachperson mit blickgeschütztem Infusionsbeutel und Infusionsbesteck.

- 1. Randomisierung und Verblindung in der Interventionsstudie

Die Probanden werden im Verhältnis 1:1 doppelblind in die Verum- oder Placebogruppe randomisiert. Dabei wird gemäss dem Ferritinwert beim Screening (≤25 μg/l oder >25 μg/l), dem Geschlecht und dem Ausmass der vorbestehenden Müdigkeit (<4 Punkte versus ≥4 Punkte auf der numerischen Messskala) stratifiziert. Die Randomisierung und Stratifizierung erfolgt durch die Pflegefachfrauen der „Clinical investigation unit“ mit Hilfe des Computers der Studiendatenbank.

Da sich das Placebo und das Verum-Präparat farblich unterscheiden, muss die Zubereitung und Verabreichung der Infusion durch eine externe Person erfolgen, die sonst nicht in der Betreuung der Probanden involviert ist. Diese Aufgabe übernehmen Pflegefachfrauen der Clinical Investigation Unit (CIU) des Inselspitals. Die Pflegefachpersonen der CIU randomisieren den Probanden am Computer, bringen die fertig zubereitete Infusion ins Blutspendezentrum und verabreichen diese. Mit Hilfe schwarz eingefärbter Infusionsschläuche und durch Abdecken des Infusionsbeutels mit Tüchern wird gewährleistet, dass weder der Proband noch das Personal des Blutspendezentrums den Studienarm herausfinden können. Eisen-Carboxymaltose ist im Vergleich zu Placebo mit leicht häufigeren Nebenwirkungen assoziiert^24^. Vor allem gastrointestinale Beschwerden und Allgemeinsymptome wurden beschrieben. Das Ausmass dieser Nebenwirkungen ist aber so gering, dass sie keinen relevanten Einfluss auf die Verblindung und damit die statistische Aussagekraft haben werden.

- 1. Vorzeitiger Studienaustritt

Der Proband kann jederzeit ohne Angabe eines Grunde aus der Studie austreten und seine Einwilligungserklärung zurückziehen. In diesem Fall werden keine weiteren Daten mehr erhoben und sämtliche Blutproben vernichtet. Bereits erhobene Daten werden bei der statistischen Auswertung der Studie jedoch mit eingeschlossen. Falls das Studienmedikament bereits verabreicht wurde, wird auf Wunsch des Probanden eine abschliessende medizinische Untersuchung durchgeführt.

- 1. Probandenbetreuung

Bei der Übersichtsstudie wird die Information der Blutspender und die Datensammlung durch das Pflegepersonal des Blutspendedienstes mitunterstützt. Die Interventionsstudie wird durch die speziell für die Studie angestellte Studienassistentin betreut. Sie arbeitet unabhängig vom Blutspendebetrieb. Sie ist verantwortlich für das Aufbieten und die Infor­mation der Probanden, Betreuung der Probanden im Blutspendezentrum und für das Ausfüllen der Frageboden. Vor Ver­abreichung des Studienpräparats kontrolliert und visiert der Spendearzt die Einverständniserklärung und beantwortet allfällige zusätzliche Fragen des Probanden zur Studie.

1. Auswahl der Versuchspersonen
   1. Übersichtsstudie

Konsekutive Blutspender am Blutspendezentrum Bern (Murtenstrasse 42, 3008 Bern) der Blutspendedienst SRK Bern AG werden anlässlich ihrer Spende dazu eingeladen, an einer Übersichtsstudie betreffend Eisenmangel teilzunehmen. Der Spender muss zu einer zusätzlichen Blutentnahme und zum Beantworten eines Fragebogens einwilligen.

- - 1. Einschlusskriterien:
- Unterschriebene Einverständniserklärung
- Spendetaugliche Vollblutspender
  - 1. Ausschlusskriterien:
- Mit Müdigkeit assoziierte aktive Krankheit oder Medikation
- Eingeschränkte Fähigkeit, die Einverständniserklärung zu verstehen
- Eingeschränkte Fähigkeit, den Fragebogen auszufüllen
- Erstspender
  1. Interventionsstudie

Alle Teilnehmer der Übersichtsstudie mit einem Serumferritin ≤50.0 μg/l, welche die Einschlusskriterien erfüllen und keine Ausschlusskriterien aufweisen, werden von der Studienkoordinatorin telephonisch zur Teilnahme an der Interventionsstudie eingeladen.

- - 1. Einschlusskriterien:
- Teilnahme an der Übersichtsstudie
- Spendetauglichkeit
- Mindestens eine frühere Blutspende
- Unterschriebene Einverständniserklärung

- Serumferritin ≤50.0 μg/l

- - 1. Ausschlusskriterien:
- Anämie: Hämoglobin <121 g/l (♀) oder <135 g/l (♂)
- Frühere Unverträglichkeitsreaktion auf intravenöses Eisen
- Durchgemachte anaphylaktische Reaktionen
- Aktive systemische Infekte jeglicher Art
- Bekannte angeborene oder erworbene Eisenüberladung (Hämochromatose)
- Hinweise auf akute oder chronische Blutung, insbesondere gastrointestinal
- Aktive Krankheit oder Medikation, die vermehrte Müdigkeit erklären würde (nach Ermessen des Studienleiters/Spendearztes)
- Aktive Krankheit oder Medikation, die eine intravenöse Eisensubstitution nicht ratsam erscheinen lässt (nach Ermessen des Studienleiters/Spendearztes)
- Körpergewicht <50 kg und >85 kg (♀) / >100 kg (♂)
- Alter <18 Jahre oder >70 Jahre
- Schwangerschaft und Stillen
- Eingeschränkte Fähigkeit, die Fragebogen auszufüllen
- Eingeschränkte Fähigkeit, die Einverständniserklärung zu verstehen

1. Messparameter
   1. Übersichtsstudie
      1. Fragebogen

Jeder Proband wird gebeten einen Fragebogen auszufüllen. Dieser besteht aus insgesamt 14 Fragen und evaluiert folgende Themen:

- Bisheriges Spendeverhalten (2 Fragen)
- Vorbestehen einer Blutarmut (2 Fragen)
- Subjektiv empfundene Müdigkeit (Numerische Messskala von 1 – 10)
- Körperliche Leistungsfähigkeit (Numerische Messskala von 1 – 10)
- Konzentrationsfähigkeit (Numerische Messskala von 1 – 10)
- Antriebsarmut (Numerische Messskala von 1 – 10)
- Depressive Verstimmtheit (Zwei numerische Messskalen von 1 – 10)
- Haarausfall
- Veränderungen der Fingernägel
- Durch Blutspende ausgelöste Müdigkeit (2 Fragen)
  - 1. Laboranalysen

Im Rahmen der routinemässigen Spendevorbereitungen wird das Hämoglobin kapillär bestimmt. Bei jedem Probanden werden ein 2.7 ml EDTA-Röhrchen und ein 10 ml Serum-Röhrchen abgenommen. Daraus wird bei jedem Probanden das Serumferritin bestimmt. Bei einer Zufallsauswahl aller Probanden (ein Fünftel der Probanden) wird zusätzlich ein komplettes Blutbild inklusive Erythrozytenindices, das CRP, der lösliche Transferrin-rezeptor, die Transferrinsättigung, das Zink-Protoporphyrin und das Serumhepcidin be-stimmt. Da die Analysen teilweise zu einem späteren Zeitpunkt „en bloc“ erfolgen, wird das Material portioniert und bei -70° C eingefroren.

- 1. Interventionsstudie
     1. Fragebogen

Die psychometrische Erfassung erfolgt mit standardisierten und auf Deutsch validierten Fragebogen im Selbstevaluationsverfahren in folgenden Dimensionen:

1) Müdigkeit (Fatigue)

2) Gesundheitsbezogene Lebensqualität

3) Psychologischer Distress / Stimmung

Die Fragebogen werden als Heft zusammengefasst dem Probanden abgegeben. Der Proband beantwortet die Fragen selbstständig, wobei die Studienkoordinatorin bei Unklarheiten zur Verfügung steht. Die Be­antwortung erfolgt im Blutspendezentrum und beansprucht ca. 30 Minuten.

Die numerische Messskala von 1 bis 10 für das Symptom Müdigkeit (Hauptendpunkt) wurde in einem Vorversuch bei 79 gesunden Blutspendern ausgetestet. Die mediane Müdigkeit lag dabei bei 3. Nur 7 von 79 gaben den tiefstmöglichen Wert 1 an. 9 von 79 quantifizierten ihre Müdigkeit mit 2 und 29 von 79 mit 3. Bei 24 von 79 lag der Wert bei 5 oder höher. Somit ist der Test gut zur Erfassung der Müdigkeit geeignet und erlaubt es fast allen Probanden, eine geringere Müdigkeit anzugeben.

Bei der zweiten Visite wird zusätzlich mit einer weiteren numerischen Messskala mit einem Spektrum von -10 bis +10 danach gefragt, ob sich für den Probanden die Müdigkeit subjektiv verändert hat (sekundärer Endpunkt).

### Angewandte Tests:

- Müdigkeitsempfinden mit numerischer Messskala von 1 bis 10^5^

- Numerischen Messskala von -10 bis +10 für die subjektiv wahrgenommene Änderung

der Müdigkeit (nur zweite Studienvisite)

- Multi-dimensional fatigue symptom inventory, short form^23^ (MFSI-SF): 30 items

- EUROQol für Lebensqualität^25^ (EQ): 6 items

- Symptom checklist SCL-27^26^ (SCL-27): 27 items

- Jenkins sleep questionnaire^27^ (JSQ): 4 items

- - 1. Laboranalysen

Sowohl bei der ersten wie bei der zweiten Visite werden je 10 ml Serum und 2.7 ml EDTA-Blut abgenommen. Daraus werden das Blutbild inklusive Erythrozytenindices (Advia 120), das CRP, das Serumferritin, die Transferrinsättigung, der lösliche Transferrinrezeptor, das Zink-Protoporphyrin und das Serumhepcidin bestimmt. Da die Analysen teilweise zu einem späteren Zeitpunkt „en bloc“ erfolgen, wird das Material portioniert und bei -70° C einge-froren. Bei Frauen im gebärfähigen Alter wird vor Studieneinschluss ein Urin-Schwanger-schaftstest durchgeführt.

1. Sicherheit
   1. Sicherheitsprofil des Studienmedikaments

Eisencarboxymaltose (Ferinject®) ist in der Schweiz seit 2008 zugelassen. Das Präparat ist dafür vorgesehen, in Arztpraxen ohne spezielle Sicherheitseinrichtungen verabreicht zu werden. Eisencarboxymaltose (Ferinject®) hat ein sehr günstiges Nebenwirkungsprofil^28^. Am häufigsten sind Reaktionen an der Injektionsstelle. Daneben finden sich milde Kopfschmerzen, Gastrointestinalbeschwerden und Hautausschläge. Anaphylaktische Reaktionen, wie sie bei früheren intravenösen Eisenpräparaten gefürchtet waren, sind bei Ferinject® eine grosse Rarität. Das Blutspendezentrum Bern wäre zur Behandlung solcher Komplikationen bestens vorbereitet. Die Dosis wurde so gewählt, dass es bei keinem Probanden zu einer Eisenüberladung kommen kann.

- 1. Definitionen

# (Siehe auch Appendix B, Seite 13)

- - 1. Adverse event (AE)

Eine Nebenwirkung im Sinne dieses Protokolls (adverse event = AE) ist jedes Auftreten oder Verschlechtern eines Symptoms, pathologischen Befundes oder einer medizinischen Krankheit während oder nach Verabreichen der Studienmedikation, unabhängig davon ob ein Zusammenhang mit dem Studienmedikament vermutet wird. Nebenwirkungen werden

mittels offener Fragen evaluiert. Falls vorhanden, folgen detailliertere und spezifischere Fragen. Eine körperliche Untersuchung, spezielle Labortests oder andere Untersuchungen zur Feststellung werden nicht routinemässig durchgeführt und werden nur angeordnet, falls das Ausmass oder die Art der Beschwerden dies medizinisch erfordern.

- - 1. Serious adverse event (SAE)

Eine ernsthafte Nebenwirkung (Serious adverse event = SAE) ist jede Nebenwirkung die

- im Tod endet
- ein lebensbedrohliches Ausmass annimmt
- eine Hospitalisation erfordert
- zu anhaltenden Behinderungen führt
- zu kongenitalen Anomalien / Geburtsdefekten ines Kindes führt
- sonstwie medizinisch bedeutungsvoll ist (gemäss Urteil des Untersuchers)
  - 1. Adverse drug reaction (ADR)

Eine unerwünschte Medikamentennebenwirkung (Adverse drug reaction = ADR) ist jede schädigende oder unerwünschte Reaktion auf das Studienmedikament, falls ein Zusammenhang mit diesem vermutet werden muss. Es kann sich um eine bekannte oder eine bisher noch nie beschriebene Nebenwirkung handeln.

- - 1. Suspected unexpected serious adverse reactions (SUSAR)

Eine erwartete unerwartet-schwere Nebenwirkung (Suspected unexpected serious adverse reaction = SUSAR) ist jede Nebenwirkung, die zwar schon beschrieben wurde, aber schwerer ausfiel, als wie es in der Medikamenteninformation beschrieben ist.

- 1. Dokumentation und Beurteilung von Nebenwirkungen

**(Siehe auch Appendix B, Seiten 14 bis 16)**

Der Schweregrad der Nebenwirkungen wird gemäss der NCI v.3.0-Kriterien klassifiziert. Alle Nebenwirkungen und alle zu deren Behandlung verabreichte Therapien werden in der Probandendokumentation und im eCRF vermerkt. Falls Nebenwirkungen bei der zweiten Studienvisite noch vorhanden sind, werden sie bis zu derem Verschwinden nachverfolgt (telephonisch, falls medizinisch verantwortbar). SAEs werden zusätzlich auf einem speziellen SAE-Formular aufgelistet. Ob ein Zusammenhang mit der Studienmedikation besteht, wird wie im Appendix B unter Punkt 4.3. beschrieben beurteilt.

- 1. Notifikation von Nebenwirkungen und jährlicher Sicherheitsbericht

**(Siehe auch Appendix B, Seiten 17 bis 35)**

Der Principal investigator als Investigator/Sponsor der Studie stellt sicher, dass ihm alle SAEs und SUSARs in der Regel gleichentags vom Studienteam gemeldet werden. Er sorgt dafür, dass bei Abwesenheiten immer ein befugter und kompetenter Stellvertreter erreichbar ist.

Lebensbedrohliche und tödliche SAEs werden sofort der Kantonalen Ethikkommission gemeldet. Die übrigen SAEs werden in einem jährlichen Sicherheitsbericht zuhanden der KEK Bern zusammengefasst. Serious Adverse Drug Reactions (SARs) und SUSARs werden gemäss den behördlichen Richtlinien an die Kantonale Ethikkommission und Swissmedic gemeldet. Das genaue Vorgehen ist im Appendix B unter Punkt 4.5.3 beschrieben.

- 1. Schwangerschaft

Bei jeder Frau im gebärfähigen Alter wird vor Studieneinschluss ein Urin-Schwanger-schaftstest durchgeführt. Schwangere Frauen können nicht in die Studie eingeschlossen werden. Alle Frauen im gebärfähigen Alter sind verpflichtet, für mindestens drei Woche eine Konzeption zu verhindern. Falls keine andauernde (medikamentöse oder mechanische) Antikonzeption angewandt wird, muss vorübergehend mit einer mechanischen Methode (Kondom) verhütet werden.

Bei der zweiten Studienvisite wird spezifisch danach gefragt, ob in der Zwischenzeit eine Schwangerschaft bekannt geworden ist (ein Schwangerschaftstest wird nicht durchgeführt). Ist dies der Fall, wird bei jeder Frau nach der Geburt der Ausgang der Schwangerschaft erfragt und im eCRF notiert.

1. Poweranalyse und Statistik

Der Hauptendpunkt der Interventionsstudie ist ein Unterschied im mittleren Müdigkeitsempfinden auf der numerischen Messskala zwischen der Ferinject- und der Placebo-Gruppe vier bis sechs Wochen nach Infusion des Studienpräparates. Aufgrund von Erfahrungswerten aus der Literatur kann von einer Standardabweichung der Müdigkeitswerte von 2.5 Punkten ausgegangen werden. Ein mittlerer Unterschied von einem Punkt auf der Skala gilt als klinisch relevant.

200 Probanden pro Gruppe führen zu einer Power von >95%, eine solche Reduktion bei einem zweiseitgen α-Fehler von 0.05 zu detektieren. Diese Fallzahl führt für eine Sekundäranalyse gleichzeitig zu zirka 85% Power, eine statistische Interaktion des Behandlungseffektes mit einer Subgruppe in der Grössenordnung von 1.5 Punkten zu detektieren. Primäres Stratifikationskriterium wird der Ferritinwert bei Baseline sein (<25 µg/l versus ≥25 µg/l). Sekundäre Stratifizierungskriterien werden Geschlecht und Müdigkeit bei Baseline sein (<4 versus ≥4 auf der numerischen Messskala).

Die Mittelwerte auf der numerischen Messskala werden mittels Kovarianz-Analyse zwi-schen den Gruppen verglichen, welche für den Baseline-Wert der numerischen Messskala korrigiert wurde. Für stratifizierte Analysen werden wir mittels least squares Regressionsmodellen einen Interaktionstest durchführen. Alle Analysen werden nach dem Intention-to-treat Prinzip durchgeführt, wobei alle randomisierten Patienten in die Analyse eingeschlossen werden, in der Gruppe, zu welcher sie initial randomisiert wurden. Allenfalls fehlende Werte werden mittels Multiple imputation ersetzt. In einer Sensitivitätsanalyse werden nur Probanden mit vollständigen Daten eingeschlossen. Alle P-Werte sind zweiseitig. Die Datenanalyse wird unter blinden Bedingungen durchgeführt, das heisst, der zuständige Statistiker wird nicht wissen, welche der beiden analysierten Gruppen Eisen und welche Plazebo erhalten hat. Alle Analysen werden in Stata Release 11 durchgeführt (StataCorp, College Station, TX).

1. Studienspezifische Vorsichtsmassnahmen

Das Studienpräparat wird generell gut vertragen. Höhergradige Nebenwirkungen sind selten. Spezielle Vorsichtsmassnahmen sind nach Verabreichung der Studienmedikation nicht indiziert. Vorausgesetzt der Proband fühlt sich unverändert wohl, ist Autofahren nach Verabreichen der Studienmedikation nicht verboten. Kommt es im Anschluss an die Studienmedikation zu Nebenwirkungen, sind die Probanden aufgefordert, sofort den Spendearzt des Blutspendezentrums Bern oder im Notfall einen Notfallarzt zu kontak-tieren. Dem Probanden wird nach Verabreichung der Studienmedikation ein entsprechender Informationszettel mit den Kontaktdaten mitgegeben.

- 1. Schwangerschaft

Es existieren keine Hinweise für eine embryonale oder fetale Toxizität von Eisen-Carboxymaltose. Da Daten fehlen, ist die Verabreichung während des ersten Trimesters aber kontraindiziert. Aus diesem Grund wird bei jeder Frau im gebärfähigen Alter (Menarche bis gesicherte Menopause) ein Urin-Schwangerschaftstest durchgeführt. Im Falle eines positiven Resultates kann die Probandin nicht in die Studie eingeschlossen werden. Die elektronische Randomisierung für die Studie ist erst möglich, nachdem ein negatives Resultat des Schwangerschaftstests ins eCRF eingetragen und visiert wurde.

Im geplanten Studiensetting ist ein Serum-Schwangerschaftstest nicht praktikabel. Da das frühschwangerschaftliche Risiko der Eisen-Carboxymaltose als sehr gering eingeschätzt werden kann (zwei Jahre nach Marktzulassung wurden keine Schwangerschaftskompli-kationen durch Eisen-Carboxymaltose berichtet) und ein Urintest bei korrekter Durchführung eine Schwangerschaft in >99% der Fälle detektiert, ist die Sicherheit durch einen Urin-Schwangerschaftstest genügend gewährleistet. Zudem darf davon ausge-gangen werden, dass auch in der ärztlichen Praxis in aller Regel Urin-Schwanger-schaftstests vor Ferinject®-Verabreichung durchgeführt werden.

Die von Swissmedic bewilligte Medikamenteninformation gibt keine Empfehlung ab, wie lange nach der Medikamentenverabreichung eine Konzeption vermieden werden muss. Im klinischen Alltag wird deshalb nach Gabe von Ferinject® nicht zwingend eine Kontrazeption angeordnet. Eisen-Carboxymaltose wird sehr rasch in die Makrophagen des retikulo-endothelialen Systems aufgenommen, wo es in Eisenhydroxid und Zuckerbestandteile abgebaut wird. Die Eliminationshalbwertszeit im Blut beträgt 7 bis 12 Stunden^28^. Danach ist Eisen an Transferrin und Ferritin gebunden, wodurch toxische Effekte verhindert werden.

Aufgrund obiger Überlegungen müssen sich Studienteilnehmerinnen im gebärfähigen Alter für eine mindestens dreiwöchige Antikonzeption nach Verabreichung der Studien-medikation verpflichten. Falls keine Dauerantikonzeption (mechanisch oder medika-mentös) durchgeführt wird, muss für drei Wochen mit mechanischen Methoden (z.B. Kondomen) verhütet werden. Bei Partnerinnen von männlichen Studienteilnehmern sind keine Antikonzeptionsmassnahmen indiziert.

1. Pflichten des Prüfers
   1. Einhaltung der gesetzlichen Bestimmungen

Die Studie wird gemäss Anforderungen für „Good Clinical Practice“ (GCP) durchgeführt. Allfällige Protokolländerungen werden vor Inkrafttreten der Kantonalen Ethikkommission Bern und Swissmedic zur Bewilligung vorgelegt. Der Principal Investigator und gleichzeitig Sponsor der Studie ist verantwortlich für die Schulung sämtlicher, an der Studie beteiligter Mitarbeiter. Er gewährleistet die Einhaltung des Studienprotokolls, der GCP-Richtlinien und der gesetzlichen Bestimmungen.

- 1. Berichterstattung

**(Siehe auch Appendix B)**

Der Principal Investigator und gleichzeitig Sponsor der Studie ist verantwortlich für die fristgerechte Meldung von schweren Nebenwirkungen (SAEs, SARs, SUSARs) an die Kantonale Ethikkommission und an Swissmedic. Die Details sind im Appendix B geregelt. Einmal pro Jahr wird ein Sicherheitsbericht verfasst. In einem Abschlussbericht werden die wichtigsten Studienresultate mittels geeigneter Grafiken und Tabellen zusammengefasst. Die Berichterstattung erfolgt unvoreingenommen objektiv. Die Berichte werden sowohl der Kantonale Ethikkommission Bern wie Swissmedic innerhalb der vorgeschriebenen Fristen zugestellt.

- 1. Versicherung

Alle Probanden sind im Rahmen des Kollektivversicherungsvertrags zwischen dem Inselspital Bern und der AXA-Winterthur Versicherung für Schäden im Rahmen der Studienteilnahme versichert.

1. Ethische Überlegungen
   1. Übersichtsstudie

In der Übersichtsstudie werden dem Probanden anlässlich einer Vollblutspende zwei zusätzliche Blutröhrchen abgenommen. Zudem muss er einen Fragebogen ausfüllen, was ca. 15 Minuten in Anspruch nimmt. Davon abgesehen erwachsen dem Probanden keine Nachteile. Die Anonymität der Daten ist gewährleistet. Die Erkenntnisse aus der Übersichtsstudie erweitern unser Wissen über das Ausmass der Eisenmangelproblematik bei regelmässigen Blutspendern und kommen dadurch allen Blutspender zugute.

Da die Probanden gemäss der Einschlusskriterien nicht an einer Anämie leiden, besteht aus medizinischer Sicht kein Handlungsbedarf Es ist daher nicht vorgesehen, jedem Studienteilnehmer die Resultate des Eisenstatus zuzuschicken. Hingegen werden die Resultate bei einer nächsten Blutspende oder auf telephonische Nachfrage hin gerne mitgeteilt.

- 1. Interventionsstudie

Eisencarboxymaltose (Ferinject®) hat ein sehr günstiges Nebenwirkungsprofil^28^. Am häufigsten sind Reaktionen an der Injektionsstelle. Daneben finden sich milde Kopfschmerzen, Gastrointestinalbeschwerden und Hautausschläge. Anaphylaktische Reaktionen, wie sie bei früheren intravenösen Eisenpräparaten gefürchtet waren, sind bei Ferinject® eine grosse Rarität. Das Blutspendezentrum Bern ist zur Behandlung solcher Komplikationen zudem gut vorbereitet. Die Eisencarboxymaltose-Dosis wurde so gewählt, dass zwar bei jedem Probanden die Eisenspeicher genügend gefüllt werden, es aber gleichzeitig nicht zu einer Eisenüberladung mit toxischen Langzeitschäden kommen kann. Rechnerisch ist nicht zu erwarte, dass das Serumferritin nach Erreichen eines Gleichgewichtszustandes über der oberen Labornorm zu liegen kommt. Zusammengefast sind die potentiellen Gefahren des Studienmedikaments sehr gering.

Die eine Hälfte der Probanden wird eine Eisensubstitution bekommen, die andere Hälfte nur Placebo (0.9% NaCl). Zwar ist es heute bei vielen Ärzten gängige Praxis, einen Eisenmangel ohne Anämie zu substituieren. Dies beruht aber nicht auf wissenschaftlichen Daten. Aus Sicht einer evidenzbasierten Medizin besteht somit weder für den Probanden im Placebo-Arm noch für denjenigen im Verum-Arm ein nachgewiesener Vorteil.

Nach Abschluss der Interventionsstudie wird jedem Probanden schriftlich mitgeteilt, ob er das Eisenpräparat oder das Placebo bekommen hat. Zudem werden die Laborresultate der ersten Studienvisite sowie der Ferritinwert anlässlich der Abschlussvisite zugesandt. Diese Informationen erlauben es dem behandelnden Hausarzt, den Patienten weiterhin korrekt zu behandeln.

Die Studie verspricht sehr relevante Resultate sowohl für Patienten wie auch gesunde Blutspender mit Eisenmangel ohne Anämie. Die Ferritin-Untergrenze, ab welcher Eisen substituiert werden muss, könnte genauer festgelegt werden und damit ermöglichen, dass künftig unnötige Eiseninfusionen vermieden werden. Sollten nachteilige Auswirkungen eines Eisenmangels auf Blutspender nachgewiesen werden können, müsste das Prozedere im Blutspendewesen angepasst werde. Die käme langfristig allen Blutspendern zugute. Aufgrund all dieser Überlegungen rechtfertig der allgemeine medizinische Nutzen der Studie das sehr geringe Risiko für den einzelnen Probanden.

1. Qualitätssicherung
   1. Monitoring

**(Siehe Appendix A: Monitoring Plan)**

Das Monitoring der Studie erfolgt durch die Clinical Trials Unit (CTU) des Inselspitals unter Einhaltung der GCP-Richtlinien. Es werden keine Mitarbeiter der CTU am Monitoring beteiligt sein, die auch sonst an der Studie mitarbeiten. Das genaue Vorgehen ist in Appendix A (Monitoringplan) festgelegt.

- 1. Inspektionen

Für Audits und Inspektionen wird der Kantonalen Ethikkommission Bern und den zuständigen Behörden Zutritt gewährleistet. Die Einsicht in alle Originaldaten sowie die elektronische Datenbank wird garantiert.

- 1. Behandlung von Daten

**(Siehe auch Appendix B, Seiten 30+31)**

Jeder Proband wird zweimal ein Fragebogenset ausfüllen. Zusätzliche medizinische Informationen werden auf ein standardisiertes Formular eingetragen. Dieses entspricht der Probandenakte (source document). Alle Daten werden in Webspirit eingegeben. Webspirit ist eine Web-basierte klinische Studiensoftware für elektronische Daten-erfassung. The Datenbank führt Validitätskontrollen durch, um Fehler oder verdächtige Ausreisser zu erkennen. Sie enthält integrierte Monitoringfunktionen einschliesslich der Generierung von Nachfragen (Queries). Die Datenbank wird zentral von der CTU Bern betreut und unterhalten.

- - 1. Case Report Forms

Alle Daten, die ins elektronische “Case report form” (eCRF) eingegeben werden, müssen in der Probandenakte (source document) hinterlegt sein. Das “Case report form” gilt nicht als “Source document”. Alle vom Protokoll vorgeschriebenen erhobenen Daten und durchgeführten Massnahmen werden im eCRF eingetragen. Die Daten werden durch die Studienassistentin ins eCRF eingegeben. Die Eingaben müssen vollständig, genau und pünktlich (innerhalb einer Woche) nach der Studienvisite erfolgen.

Der Principal Investigator oder ein von ihm bevollmächtigter Stellvertreter kontrollieren die Eingaben stichprobenweise auf Genauigkeit und Übereinstimmung mit dem Protokoll und signieren das eCRF, wenn es vollständig ist. Nach Vorankündigung kann die Übereinstimmung der Rohdaten (source data) und der Einträge im eCRF durch den Monitor oder die zuständigen Behörden kontrolliert werden. Der Principal Investigator garantiert die Identität der Rohdaten und der Einträge im eCRF.

- - 1. Archivierung

Die endgültigen “Case report forms” werden ausgedruckt und in einem Studienordner zusammen mit den anderen Dokumenten (z.B. dem Protokoll) an einem sicheren, verschlossenen Ort aufbewahrt. Der Principal Investigator ist für die langfristige Archivierung verantwortlich.

- - 1. Elektronische Datenbank

Die Infrastruktur der elektronischen Datenbank wird von der CTU Bern zur Verfügung gestellt. Eingaben können Web-basiert auf lokalen Computern der beteiligten Institutionen erfolgen. Die Studiendaten werden in einer relationalen SQL-Datenbank unter Benutzung des Webspirit-Systems der CTU Bern gespeichert.

Die Datenbank-Server stehen in einem verschlossenen, geschützten Raum. Nur die Systembetreuer haben Zugang zum Server. Ein Back-up wird täglich erstellt und wird in einem anderen Gebäude aufbewahrt. Ein zweites Back-up wird auf einem Server im Institut für Sozial und Präventivmedizin aufbewahrt. Befugte Benutzer können die Datenbank mittels eines persönlichen Passwort bedienen.

- - 1. Datenvalidierung

Ein mehrstufiger Validierungsplan garantiert die Korrektheit und Konsistenz der Daten. Die Daten können nur nach einer Überprüfung auf Vollständigkeit und Plausibilität gespeichert werden. Die Dateneingabe erfolgt übers Internet mit Masken, die die Konsistenz der Daten überprüfen. Daten werden auch mit früher eingegebenen Daten auf Übereinstimmung überprüft. Eine nachträgliche Anpassung der Daten wird elektronisch festgehalten. Die Identität des Probanden wird durch eine Fall-ID gewährleistet. Diese ID wird dem Probanden von der Datenbank automatisch zugeordnet. Es besteht keine logische Verbindung zwischen der Fall-ID und dem Namen des Probanden. Der Datenaustausch findet ausschliesslich unter Benutzung der Fall-ID statt.

- - 1. Datenanalyse und Archivierung

Für die Auswertung werden die Daten in eine statistische Software überführt. Zu diesem Zeitpunkt wird die Datenbank in eine Archivform überführt, die später nicht mehr geändert werden kann. Alle Studiendaten in Papierform werden unter der Verantwortung des Principal Investigator in Stahlbehältern für 10 Jahre aufbewahrt. Die SQ-Datenbank wird durch die CTU ebenfalls für 10 Jahre aufbewahrt. Die CTU Bern wird Interim- und Schlussberichte sowohl in elektronischer wie in Papierform aufbewahren.

- 1. Umgang mit Blutproben

Nicht alle vorgesehenen Analysen werden sofort durchgeführt. Verschiedene Analysen erfolgen erst zu einem späteren Zeitpunkt. Zu diesem Zweck wird Serum aliquotiert und bei -70° C eingefroren. Die Lagerung erfolgt im Routinelabor des Blutspendedienstes Bern an der Murtenstrasse 133, 3008 Bern. Der Gefrierschrank ist stets verschlossen. Spätestens ein Jahr nach Abschluss der Studie werden alle Proben vernichtet. Es werden keine Analysen durchgeführt, die nicht im Protokoll vorgesehen sind.

1. Zusatzinformationen für die Notifizierung (Swissmedic)
   1. Drug Accountability

Die Lieferung des Studienmedikaments erfolgt direkt an die Clinical Investigation Unit (CIU) des Inselspitals und wird dort abgeschlossen, für Unbefugte unerreichbar und bei Raumtemperatur (+4° C bis 25° C) gelagert. Der Eingang der Medikamente wird mit Lot-Nummer und Verfalldatum auf dem Formular „Medikamentenlieferungen“ vermerkt und von einer CIU-Pflegefachperson signiert.

Jede Entnahme von Ferinject®-Ampullen aus dem entsprechend beschrifteten Lager in der CIU wird auf dem „Medikamenten-Log-Formular“ der ISUB-Studie vermerkt und von der entsprechenden CIU-Pflegefachperson visiert. Festgehalten wird Name und Geburtsdatum des Probanden, die Anzahl und Grösse der entnommenen Ampullen, die Lot-Nummer und das Verfalldatum.

- 1. Erfassung der Compliance

Die intravenöse Verabreichung des Studienmedikaments erfolgt unter der Aufsicht einer Pflegefachperson der CIU am Tag des Studienbeitritts (d.h. unmittelbar nach Unterschrift der Einverständniserklärung). Probleme mit Malcompliance sind deshalb nicht zu erwarten. Sollte die Infusion aus irgend einem Grund nicht verabreicht werden können, wird dies durch die CIU-Pflegefachperson auf dem Case Report Form, im eCRF und auf dem Medikamenten-Log-Formular der Studie vermerkt

- 1. Beschriftung des Prüfpräparates

Von der Firma Vifor Pharma SA wird das kommerzielle Präparat Ferinject® in der üblichen Verpackung und normal beschriftet geliefert. Die Verpackung und die Ampullen enthalten eine Lot-Nummer und das Verfalldatum.

Die Infusion wird durch eine Pflegefachperson der CIU gemäss der Computer-Randomi-sierung zubereitet. Dies erfolgt in den Räumlichkeiten der CIU unter sterilen Bedingungen. Der Infusionsbeutel (Verum oder Placebo) wird mit einer bedruckten Etikette beschriftet, welche die Probandennummer, den Studiennamen (ISUB-Studie), den Principal investigator/Sponsor (Universitätsklinik für Hämatologie, Inselspital Bern), das Herstellungsdatum und die Herstellungszeit enthält. Die Randomisierung (Verum versus Placebo) ist auf der Etikette nicht vermerkt.

Die Infusion muss unmittelbar nach Zubereitung (maximal 120 Minuten) erfolgen. Für den Transport der fertig zubereiteten Infusion von CIU ins Blutspendezentrum (ca. 200 Meter zu Fuss auf öffentlichen Strassen) wird der Infusionsbeutel in einen sauberen Beutel verpackt und die ganze Infusion in einem dicht abschliessbaren Behälter transportiert. Die Infusion ist dabei immer unter Aufsicht der Pflegefachfrau der CIU, die die Infusion im Blutspendezentrum verabreichen wird.

- 1. Publikation der Resultate

Die Resultate der Studie werden in mindestens einer Fachzeitschrift veröffentlicht. Alle formalen Präsentationen und Publikationen von Daten dieser Studie erfolgen als Gemeinschaftsmitteilungen aller wissenschaftlichen Mitarbeiter. Bei der ersten Publikation ist Dr. P. Keller Erstautor und Dr. S. Fontana Letztautor. Die weiteren Mitautorschaften werden gemäss wissenschaftlichem Beitrag zur Studie festgelegt und sollen auf einstimmigem Einverständnis aller wissenschaftlichen Mitarbeiter beruhen. Weitere Analysen von Daten und Publikation von Resultaten (wissenschaftliche Artikel, Vorträge) dürfen ausschliesslich unter gemeinsamer Zustimmung von Dr. P. Keller und Dr. S. Fontana erfolgen.

1. Literatur

1. Andrews NC. Disorders of iron metabolism. N Engl J Med. 1999;341:1986-1995.

2. Brownlie Tt, Utermohlen V, Hinton PS, Giordano C, Haas JD. Marginal iron deficiency without anemia impairs aerobic adaptation among previously untrained women. Am J Clin Nutr. 2002;75:734-742.

3. Bruner AB, Joffe A, Duggan AK, Casella JF, Brandt J. Randomised study of cognitive effects of iron supplementation in non-anaemic iron-deficient adolescent girls. Lancet. 1996;348:992-996.

4. Patterson AJ, Brown WJ, Roberts DC. Dietary and supplement treatment of iron deficiency results in improvements in general health and fatigue in Australian women of childbearing age. J Am Coll Nutr. 2001;20:337-342.

5. Verdon F, Burnand B, Stubi CL, et al. Iron supplementation for unexplained fatigue in non-anaemic women: double blind randomised placebo controlled trial. Bmj. 2003;326:1124.

6. Der Iron-Code. Coop-Zeitung vom 6 November 2007; #45.

7. Skikne B, Lynch S, Borek D, Cook J. Iron and blood donation. Clin Haematol. 1984;13:271-287.

8. Newman B. Iron depletion by whole-blood donation harms menstruating females: the current whole-blood-collection paradigm needs to be changed. Transfusion. 2006;46:1667-1681.

9. Raftos J, Schuller M, Lovric VA. Iron stores assessed in blood donors by hematofluorometry. Transfusion. 1983;23:226-228.

10. Simon TL, Garry PJ, Hooper EM. Iron stores in blood donors. Jama. 1981;245:2038-2043.

11. Pedersen NS, Morling N. Iron stores in blood donors evaluated by serum ferritin. Scand J Haematol. 1978;20:70-76.

12. Finch CA, Cook JD, Labbe RF, Culala M. Effect of blood donation on iron stores as evaluated by serum ferritin. Blood. 1977;50:441-447.

13. Milman N, Sondergaard M. Iron stores in male blood donors evaluated by serum ferritin. Transfusion. 1984;24:464-468.

14. Frey BM, Rüfer A, Hardegger K, et al. Iron Balance Remains Stable in Life-Long Blood Donors (Poster DGTI 2007). Transfus Med Hemother. 2007;34 (Suppl 1):69.

15. Mast AE, Foster TM, Pinder HL, et al. Behavioral, biochemical, and genetic analysis of iron metabolism in high-intensity blood donors. Transfusion. 2008;48:2197-2204.

16. Schorer G, Brittenham G, Darnuzer R, Westerman M, Frey BM. Sequential blood donation decreases total body iron stores and serum hepcidin (Poster SGIM/SGH 2009). Schweiz Med Forum. 2009;9 (Suppl 46):79 S.

17. Rosvik AS, Hervig T, Wentzel-Larsen T, Ulvik RJ. Iron status in Norwegian blood donors: comparison of iron status in new blood donors registered in 1993-1997 and in 2005-2006. Vox Sang. 2009;96:49-55.

18. Ochmann O, Hardegger K, Frey B. Iron Balance in Young Blood Donors: Is the Latent Iron Deficiency Syndrome (LIDS) Universal? (Poster DGTI 2007). Transfus Med Hemother. 2007;34 (Suppl 1):71.

19. Radtke H, Tegtmeier J, Rocker L, Salama A, Kiesewetter H. Daily doses of 20 mg of elemental iron compensate for iron loss in regular blood donors: a randomized, double-blind, placebo-controlled study. Transfusion. 2004;44:1427-1432.

20. Maghsudlu M, Nasizadeh S, Toogeh GR, Zandieh T, Parandoush S, Rezayani M. Short-term ferrous sulfate supplementation in female blood donors. Transfusion. 2008;48:1192-1197.

21. Magnussen K, Bork N, Asmussen L. The effect of a standardized protocol for iron supplementation to blood donors low in hemoglobin concentration. Transfusion. 2008;48:749-754.

22. Pedrazzini B, Waldvogel S, Cornuz J, et al. The impact of iron supplementation efficiency in female blood donors with a decreased ferritin level and no anaemia. Rationale and design of a randomised controlled trial: a study protocol. Trials. 2009;10:4.

23. Stein KD, Jacobsen PB, Blanchard CM, Thors C. Further validation of the multidimensional fatigue symptom inventory-short form. J Pain Symptom Manage. 2004;27:14-23.

24. Bailie GR, Mason NA, Valaoras TG. Safety and tolerability of intravenous ferric carboxymaltose in patients with iron deficiency anemia. Hemodial Int;14:47-54.

25. Rabin R, de Charro F. EQ-5D: a measure of health status from the EuroQol Group. Ann Med. 2001;33:337-343.

26. Hardt J, Egle UT, Brahler E. [The symptom checklist-27 in Germany]. Psychother Psychosom Med Psychol. 2006;56:276-284.

27. Jenkins CD, Stanton BA, Niemcryk SJ, Rose RM. A scale for the estimation of sleep problems in clinical research. J Clin Epidemiol. 1988;41:313-321.

28. Ferinject Fachinformation. Arzneimittel-Kompendium der Schweiz. 2010; Band 1: Seiten 1700 -1702.
