# Supplementary material for: The effects of intravenous iron supplementation on fatigue and general health in non-anemic blood donors with iron deficiency: a randomized placebo-controlled superiority trial
Source: Sci Rep. 2020 Aug 26;10:14219. doi: 10.1038/s41598-020-71048-0 (PMC7449957; doi:10.1038/s41598-020-71048-0)
Supplement: Supplementary file 3 — Supplementary Information 3. [file 41598_2020_71048_MOESM3_ESM.docx]

**Supplementary File:**

English translation of the original German version of the Study protocol which was approved by Swissmedic of Switzerland.

“Swissmedic is the national authorisation and supervisory authority for drugs and medical products. The agency ensures that only high-quality, safe and effective medical products are available in Switzerland, thus making an important contribution to the protection of human and animal health.”

**
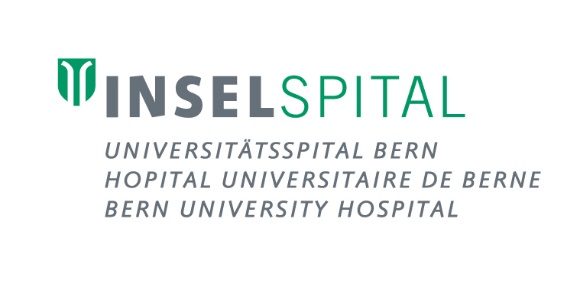
**

**Blutspendedienst SRK Bern AG**

**Blutspendezentrum Bern**

**Universitätsklinik für Hämatologie**

**Murtenstrasse 42**

**3008 Bern**

**Significance of iron deficiency for blood donation:**

**Effect of iron substitution on fatigue and general well-being in healthy blood donors**

**[ISUB : Iron substitution in blood donors]**

**Version #1.2 of 08/08/2011**

**Principal investigator: Dr. Peter Keller, M.D.**

Department of Hematology and Central Hematological Laboratory, Inselspital / University Hospital, 3010 Bern

Date: Signature:

…………………………………….…. ……………………………………………………….

**Co-investigator: Dr. med. Stefano Fontana**

Blood Donation Service SRK Bern AG, 3008 Bern

**Sponsor:** **Department of Hematology**

Dr. P. Keller, Inselspital Bern

**Monitoring:** **Clinical Trials Unit**

Prof. P. Jüni, Inselspital Bern

Other staff and institutions involved:

Prof. Dr. med. P. Jüni

Institute for Social and Preventive Medicine, University of Bern

Prof. Dr. med. R. von Känel

Chief Physician for Psychosomatic Medicine, Inselspital Bern

Prof. Dr. med. B. Lämmle

Director, University Clinic for Haematology, Inselspital Bern

Dr. Ch. Niederhauser, Blood Donation Service SRK Bern AG, 3008 Bern

Blood Donation Center Bern with employees

Murtenstrasse 42, 3008 Bern, Blood Donation Service SRK Bern AG

Clinical Trials Unit with staff, headed by Prof. P. Jüni, Inselspital Bern

Clinical Investigation Unit with staff

Direction Prof. H.P. Marti, Inselspital Bern

**Table of Contents**

1. Summary 4

2. Background and current state of knowledge 5

2.1. Iron: Physiological aspects 5

2.2 Iron deficiency without anaemia 5

2.3 Iron deficiency in blood donation 6

2.4 Study medication 7

3. Objectives and purpose 8

3.1 Fundamentals of the study concept 8

3.2 Study objectives 8

3.3 Question 9

3.3.1 Survey study 9

3.3.2 Main question of the intervention study 9

3.3.3 Secondary study questions 9

3.4. Hypothesis 9

4. Study design 10

4.1 Overview 10

4.2 Study scheme 10

4.3 Endpoints 11

4.3.1 Survey study 11

4.3.2 Intervention study 11

4.4 Procedure of the survey study 11

4.5 Procedure of the intervention study 12

4.5.1 Enrollment / first study visit 12

4.5.2 Second study visit / study completion 12

4.5.3 Study medication 13

4.6 Randomisation and blinding in the intervention study 13

4.7 Premature study termination 13

4.8 Care of the participants 14

5. Selection of study participants 15

5.1 Survey study 15

5.1.1 Inclusion criteria 15

5.1.2 Exclusion criteria 15

5.2 Intervention study 15

5.2.1 Inclusion criteria 15

5.2.2 Exclusion criteria 15

6. Measurements 16

6.1 Survey study 16

6.1.1 Questionnaire 16

6.1.2 Laboratory analyses 16

6.2 Intervention study 16

6.2.1 Questionnaires 16

6.2.2 Laboratory analyses 17

7. Safety 18

7.1 Safety profile of the trial drug 18

7.2 Definitions 18

7.2.1 Adverse event (AE) 18

7.2.2 Serious adverse event (SAE) 18

7.2.3 Adverse drug reaction (ADR) 18

7.2.4 Suspected unexpected serious adverse reactions (SUSAR) 18

7.3 Documentation and evaluation of side effects 19

7.4 Notification of adverse reactions and annual safety report 19

7.5 Pregnancy 19

8. Power analysis and statistics 20

9. Study specific precautions 21

9.1. Pregnancy 21

10. Duties of the auditor 22

10.1 Compliance with legal requirements 22

10.2 Reporting 22

10.3 Insurance 22

11. Ethical considerations 23

11.1 Survey study 23

11.2 Intervention study 23

12. Quality assurance 24

12.1 Monitoring 24

12.2 Inspections 24

12.3 Handling of data 24

12.3.1 Case Report Forms 24

12.3.2 Archiving 24

12.3.3 Electronic database 25

12.3.4 Data validation 25

12.3.5 Data analysis and archiving 25

12.4 Handling blood samples 25

13. Additional information for the notification (Swissmedic) 26

13.1 Drug Accountability 26

13.2 Recording compliance 26

13.3 Labelling of the IMP 26

13.4 Publication of the results 27

14. Literature 28

1. **Summary**

**Background:**

Deep iron stores in blood donors are a common phenomenon. However, whether iron deficiency has an effect on the health of the donors has not been sufficiently studied. The threshold values for a clinically relevant iron deficiency are very controversial both in general medicine and especially among blood donors. Some poorly controlled studies with oral iron replacement in regular blood donors have shown that the long-term ability and willingness to donate blood could be improved. However, the studies were unable to demonstrate a positive effect on the health of the donors. It is therefore still controversial today whether iron deficiency in regular blood donors should be sought and substituted.

**Aim of the project:**

- Detailed description of the iron deficiency problem in a large group of blood donors.
- Clarification of the link between low iron stores and potentially related complaints, especially fatigue.
- Definition of a lower iron storage limit in blood donors, above which somatic and psychological complaints occur.
- Proof of the effectiveness of intravenous iron substitution and thus formal proof of the connection between iron deficiency and symptoms.
- Proof of the safety of intravenous iron substitution in blood donors.

**Study design:**

- Descriptive survey in a large collective of blood donors to determine the iron status and associated symptoms
- Double-blind, randomized, placebo-controlled interventional study with intravenous administration of iron carboxymaltose (Ferinject®) or placebo in healthy blood donors with reduced to low normal iron stores

**Primary endpoint of the intervention study:**

Improvement in subjectively perceived fatigue after intravenous iron supplementation in randomized, double-blind comparison with placebo

**Main inclusion criteria of the intervention study:**

Blood donor suitability, serum ferritin ≤50.0 μg/l

**Main exclusion criteria of the intervention study:**

Anemia, intravenous iron intolerance, previous anaphylactic response, active diseases, known iron overload, acute or chronic bleeding, body weight <50 kg and >85 kg (women) / >100 kg (men), age <18 or >65 years

**2. Background and current state of knowledge**

**2.1. Iron: Physiological aspects**

The human body contains about 3 to 4.5 grams of iron, depending on sex and size. Most of this is found as haem-bound iron in the erythrocytes^1^. One gram of haemoglobin contains 3.4 mg iron. This shows that a whole blood donation of 450 ml removes between 180 and 250 mg iron from the body. In addition to its vital role as an oxygen carrier in the red blood pigment, iron is also responsible for numerous essential functions in many other proteins and enzymes. The basis of this important biological role is the high redox potential of iron. However, the potentially toxic properties of excess iron in the body are also based on the same mechanism, as increased radical formation can lead to damage to various organs. The distribution of iron in the organism is therefore well regulated. In the short term, excess iron is absorbed by macrophages of the reticuloendothelial system. In the long term, iron is stored in the cells mainly as a macro-complex with the protein ferritin. Serum ferritin therefore reflects the body's storage iron and serves in medicine as the most important laboratory parameter for the diagnosis of iron deficiency.

**2.2 Iron deficiency without anaemia**

The full picture of a severe iron deficiency is unmistakable with all the effects of anaemia such as paleness, weakness, exhaustion, tiredness and many other symptoms. Much more difficult to classify are mild iron deficiency conditions that have not yet led to anaemia. This topic is still highly controversial in internal medicine. Many different symptoms such as chronic fatigue, concentration disorders, exhaustion, depression or lack of drive are attributed to this constellation. Since iron plays a central role in many essential enzymes, for example in the muscles and the brain, such complaints can be explained theoretically well.

Clinical studies provide certain indications that iron deficiency without anaemia actually leads to symptoms. For example, after six weeks of oral iron supplementation in non-anaemic women with laboratory iron deficiency under endurance training, a significantly higher increase in aerobic capacity was demonstrated^2^. In adolescent US women with iron deficiency without anaemia, eight weeks of oral iron replacement resulted in significant improvement in various cognitive functions compared to the placebo control group^3^. In another study, dietary iron supplementation led to a general improvement in various health characteristics^4^. In an important study from the University Hospital of Lausanne, women with latent iron deficiency who suffered from unexplained fatigue were treated with placebo-controlled peroral iron. Using a numerical rating scale, a significant improvement in fatigue was shown^5^. However, this study is controversial, as due to peroral iron substitution with black discolouration of the stool and gastrointestinal complaints, the verum and the placebo could not be administered really blindly and a placebo effect could therefore not be reliably excluded.

Complaints caused by iron deficiency without anaemia are generally mild and unspecific. They can easily be confused with symptoms of other diseases or everyday ailments. It is often difficult for the doctor to decide whether

a postulated iron deficiency is actually responsible for the patient's symptoms. The condition of iron deficiency without anaemia has been propagated in recent years, especially by the pharmaceutical industry, as an independent clinical picture. Self-proclaimed iron experts in specialised medical practices ("iron centres") have also taken up the subject and practise generous intravenous iron substitution. Reports in the lay press have caused considerable unrest in broad sections of the population^6^.

The scientific evidence for generous iron substitution in iron deficiency without anaemia is insufficient. By promoting high iron limits and frequent iron infusions, negative health consequences cannot be ruled out for a large number of people. Based on the current data, it is unclear above which limit value of the iron parameters a clinically relevant iron deficiency can be excluded and no iron substitution is indicated.

**2.3 Iron deficiency in blood donation**

In blood donors, deep iron stores are a well-known phenomenon^7^. It is generally accepted that repeated donations cause or aggravate iron deficiency^8^. A pooled analysis^7^ of four studies^9-12^ found an average ferritin of 124 μg/l in first-time male donors and 47μg/l in repeat male donors. In women, the corresponding ferritin values were 46 μg/l and 25 μg/l. The development of an iron deficiency seems to depend not so much on the total number of donations, but on their frequency over time^10,13^. Data from the Zurich blood donor service SRK for long-term blood donors also showed that the iron balance stabilises in the long term with very frequent donations^14^. A study with so-called super donors (13 donations per 2 years)^15^ proves that the organism is able to massively increase the absorption of iron from the intestine during frequent blood donations. Hepcidin, which prevents enteral iron absorption, was significantly reduced in these subjects. Similar results are also reported by the Zurich blood donation service SRK^16^.

Most data on iron status in blood donors come from small, poorly controlled studies and are sometimes contradictory. In an early study from Albuquerque, NM, with 1021 unselected blood donors, serum ferritin of less than 12 μg/l^10^ was found in 23.4% of the women and 7.7% of the men. In a Norwegian study 21.7% of the female and 4.6% of the male new donors were already anaemic (haemoglobin below the donor threshold)^17^. 56.9% of these women had ferritin <30 μg/l and 20% <15 μg/l. Only in 3.3% of these men the ferritin was <30 μg/l and only in one below 15 μg/l^17^. For Switzerland, there are data published as posters from the Zurich blood donation service SRK^18^: In 1,539 donors younger than 40 years, 25.9% had ferritin below 15 μg/l. This was the case in 51.7% of women and 4.7% of men. Interestingly, even in 25.1% of healthy new donors the ferritin was less than 15 μg/l.

In the field of blood donation, it is also unclear today whether iron depletion actually has health effects. It can be proven that peroral iron substitution can keep the total body iron constant in men who frequently donate blood and even slowly increase it in women^19^. It has also been shown that peroral iron substitution can improve the ability to donate and prevent donor loss^20,21^ but there is no clear evidence that iron deficiency in blood donors really correlates with symptoms and

iron substitution can reduce complaints or adverse health outcomes. Finally, it is unclear above which lower ferritin limit iron deficiency becomes symptomatic and must be treated. Should it actually be proven that empty iron stores have a negative effect on the health of the donors, this would have direct consequences for blood donation practice. Regular monitoring of iron stores should be declared mandatory and, if necessary, longer donation intervals or regular iron replacement should be introduced.

**2.4 Study medication**

Iron carboxymaltose (Ferinject®) has been approved in Switzerland for intravenous iron replacement by Swissmedic since 2008 and is subject to health insurance contributions. The indication is for laboratory-proven iron deficiency conditions that do not respond to oral iron substitution or for which oral substitution is contraindicated for medical reasons. In the present study, only subjects are included in whom iron deficiency is very likely to occur with a ferritin ≤50 μg/l. As discussed in chapter 3.1, parenteral administration must be preferred to oral iron substitution for technical study reasons. The use of Ferinject® is intended for the pathophysiological study of the not yet fully understood phenomenon of iron deficiency without anaemia. Therefore, only the endpoints specific to the study will be assessed in terms of response. In addition, the spectrum of side effects of Ferinject® in healthy blood donors will be described.

The iron carboxymaltose infusion will be used to replenish iron stores. There is no additional iron deficit due to haemoglobin-bound iron in our volunteers. Roughly estimated, the iron stores in a woman are 500 mg and in a man 1000 mg of iron. A unit dose of 800 mg iron carboxymaltose was chosen for the study. This dose guarantees a sufficient filling of the iron stores in both sexes and in light and heavy persons. At the same time, the chosen dose should not increase the ferritin in equilibrium above the upper laboratory norm in any subject. Thus, long-term toxicity due to iron overload is not to be feared.

**3. Objectives and purpose**

**3.1 Fundamentals of the study concept**

The clinical effects of iron deficiency without anaemia are difficult to assess because they overlap with many other somatic diseases or psychological complaints. Because of their gradual onset, they may go unnoticed for a long time. The perception of iron deficiency can also vary greatly depending on the personality structure of the patient. In the group of blood donors, who are highly motivated and positive compared to the average population, one could expect that complaints are noticed rather late.

A very sensitive method is required to measure the mild and unspecific symptoms of iron deficiency. The influence of inter-individual and subjective differences should be minimized as much as possible. The recording of iron deficiency-associated complaints with the help of a questionnaire alone would therefore hardly be successful. By randomized and blinded administration of iron or placebo and measurement of subjectively perceived fatigue before and after substitution of iron deficiency, these problems can be largely avoided. The good sensitivity of a numerical rating scale for quantifying the perception of fatigue is well established^5^.

Our study concept is able to avoid numerous difficulties associated with iron deficiency-associated complaints. Only the choice of intravenous iron substitution allows reliable blinding, since peroral iron preparations are always recognized by the volunteer due to their gastrointestinal side effects. Intravenous administration also solves the problem of poor drug intake, which is unavoidable due to the gastrointestinal side effects of peroral iron^19^. The intravenous infusion leads to a rapid replenishment of the iron stores. An improvement in the patient's state of health is thus much more noticeable, which significantly increases the sensitivity of the examination. Both problems are likely to play a much greater role in a similar study^22^ currently underway in Lausanne with peroral iron.

The design of our study allows the best possible blinding between verum and placebo medication. This is absolutely relevant in the difficult complex of symptoms associated with iron deficiency.

**3.2 Study objectives**

In a survey of all blood donors at our Blood Donation Centre in Bern, the extent and frequency of iron deficiency in blood donors is to be clearly recorded. The subjectively reported complaints are to be correlated with the different iron parameters and the previous donation behaviour of the test persons.

In an intervention study with intravenous iron substitution in double-blind comparison with a placebo preparation, it is to be investigated whether the subjectively felt fatigue and other general symptoms improve and thus health effects of iron deficiency can be proven without anaemia.

**3.3. Question**

**3.3.1 Survey study**

Description of the frequency and effects of iron deficiency in a large collective of blood donors.

**3.3.2 Main question of the intervention study**

Can an improvement in subjectively perceived fatigue be demonstrated by intravenous substitution of iron in blood donors without anaemia?

**3.3.3 Secondary study questions**

Do study participants subjectively feel a change in their level of fatigue after intravenous iron substitution?

Can an improvement in general and psychological well-being after intravenous iron replacement be demonstrated by means of various psychometric tests?

Can an increase in haemoglobin and an in iron stores be demonstrated after intravenous iron substitution?

How is intravenous iron replacement tolerated iby healthy blood donors?

Can an improvement in subjectively perceived fatigue be observed in the following subpopulations: Women, men, ferritin <25 μg/l, ferritin >25 μg/l, pre-existing fatigue on the numerical rating scale <4 or >4?

**3.4 Hypothesis**

Even a slight iron deficiency that has not yet led to anaemia can cause a relevant impairment of the general condition in some people. However, the complaints are so mild and unspecific that they can only be proven by comparing the condition before and after iron replacement and carefully avoiding a subjective bias.

**4. Study design**

**4.1 Overview**

The project consists of two parts, a survey study in a blood donor collective and an intervention study with intravenous iron administration in donors with ferritin levels ≤50 μg/l.

As a screening for the intervention study, all blood donors who consent to the study will be prospectively measured for about 3 months and a questionnaire on the donor history and symptoms of iron deficiency will be handed in for completion.

The main part of the project is a randomised, double-blind, placebo-controlled study with intravenous administration of iron carboxymaltose (Ferinject®) in male and female blood donors with reduced or low normal iron stores. The primary endpoint is an improvement in the level of subjectively perceived fatigue.

**4.2 Study scheme**

**First blood donation**

**Inclusion**

**Interventionstudy**

**Final visit, second blood donation**

6-8 weeks (42-56 days)

4 to 6 weeks

**Survey study blood donor cohort**

Capillary hemoglobin; blood sample: Ferritin, others (see text)

Questionnaire „Significance of iron deficiency for blood donation”

**End of study**

**Completion of questionnaires**

Blood sample: blood count (Advia 120), ferritin, Others (see text, reserved serum -70° C)

**Selection of participants (ferritin ≤50 μg/l)**

Invitation per phone to participate in the study

**Written informed consent**

**Randomisation** (with stratification)

**Completion of questionnaires**

Blood sample: blood count (Advia 120), ferritin, Others (see text, reserved serum -70° C)

**Placebo**

Intravenous infusion for 15 min

**Ferinject**

Intravenous infusion for 15 min

**4.3 Endpoints**

**4.3.1 Survey study**

The frequency and severity of iron deficiency as well as the subjective complaints associated with iron deficiency are recorded for blood donors at the Blood Donation Centre Bern. In addition, the postulated relationship between iron deficiency, clinical symptoms and previous blood donation behaviour will be investigated. To determine the iron status, several iron parameters will be measured and the diagnostic efficiency of the different parameters will be compared.

**4.3.2 Intervention study**

**Primary endpoint:**

- Difference in mean fatigue levels on a numerical rating scale of 1 to 10 between the ferinject and placebo groups four to six weeks after study drug infusion.

**Secondary endpoints:**

- Subjectively perceived change in fatigue after administration of the study drug, measured with a numerical rating scale from -10 to +10
- Improvement in general and mental well-being after intravenous iron replacement, measured by multiple psychometric tests.
- Change in haemoglobin and iron parameters after intravenous iron substitution.
- Tolerance of intravenous iron in healthy blood donors.
- Improvement of fatigue in the following subpopulations: Women, men, ferritin <25, ferritin >25, fatigue scale <4, fatigue scale >4 (see stratification criteria)

The main endpoint "fatigue" should be asked in a colloquial sense without further definition. In an earlier study, the measurement of fatigue using a numerical rating scale or a visual-analogue scale was successfully applied to women with chronic fatigue and reduced body travel^5^. With the help of this endpoint, it was possible to demonstrate a clinically relevant reduction in subjectively perceived fatigue in a statistically significant manner. Since "fatigue" is experienced by different people in very different ways, various subqualities of "fatigue" are also recorded as a secondary endpoint with the help of the Multi-dimensional fatigue symptom inventory, short form (MFSI-SF).^23^

**4.4 Procedure of the survey study**

During the recruitment phase for the intervention study (approx. 3 months), all consecutive whole blood donors of the Blood Donation Center SRC Bern are invited to participate in a study to examine their iron status. For this purpose, each donor is given an information sheet and a consent form together with the donation questionnaire. If the donor gives his or her written consent, a 2.7 ml EDTA tube and a 10 ml serum tube are also collected before the blood donation. The serum ferritin is then determined. In a random selection, the blood count and other iron parameters are determined.

The participant is asked to complete a questionnaire that includes questions about the donor's history and iron deficiency related complaints. It takes about 10 to 15 minutes to complete the questionnaire. Information on the medical history and current medication is taken from the donor documents by the study assistant.

If the participant does not participate in the intervention study and the ferritin is very low (≤50 μg/l), she/he will be informed about the result in writing. Otherwise, she/he can ask for the result by telephone or at the next donation.

**4.5 Procedure of the intervention study**

**4.5.1 Enrollment / first study visit**

Donors with a serum ferritin ≤50 μg/l qualify for the study. Potential participants are continuously selected and invited by telephone to participate in the study, taking into account the inclusion and exclusion criteria. With preliminary consent, the participant information is sent and the candidate is invited to the first study visit four to six weeks after her/his blood donation.

On the occasion of the first visit, the donor physician will provide the information. If the donor provides definite consent to participate in the study with a signed declaration of consent, the volunteer is given the questionnaire package to fill out and a blood sample is taken. From this, the blood count including erythrocyte indices (Advia 120), serum ferritin and other iron parameters are determined. Since some of the analyses are carried out later, serum is portioned and frozen at -70° C. In women of childbearing age, a urine pregnancy test is taken.

As soon as the participant has signed the consent, the nursing staff of the Clinical Investigation Unit (CIU) is informed. They randomize and stratify the patient using the study database and prepare the study medication. The finished infusion is packaged so that its color cannot be recognized. The infusion is taken to the blood donation centre and administered to the patient by the CIU nurse. In order to avoid paravenous invasions with skin discoloration, which could also affect blindness, the study drug is infused exclusively through an indwelling venous cannula (Venflon).

**4.5.2 Second study visit / study completion**

In order not to blur the effect of iron substitution, no whole blood, red cells, thrombocytes or plasma may be donated from the first blood donation at the screening until the final visit. The second, final visit takes place six to eight weeks after administration of the study drug (allowed time window 42 to 56 days). The participant is asked to complete the questionnaire package for a second time. In addition, the study coordinator will ask about any side effects on the study medication. Blood is then sampled and the blood count and various iron parameters are determined again. The material is frozen at -70° C. The study is now completed for the participant. If she or he wishes, a regular blood donation may be made at the end of the study.

Following this visit, the participant is informed in writing whether he or she has received the iron supplement or the placebo. In addition, the laboratory results of the first visit and the ferritin value of the second visit will be sent to the volunteer.

**4.5.3 Study medication**

Iron carboxymaltose (Ferinject®)

Iron carboxymaltose has been authorised in Switzerland by Swissmedic since 2008 for the treatment of iron deficiency. The drug is provided free of charge by the company Vifor Pharma Ltd. It is administered in a 0.9% NaCl carrier solution as an intravenous infusion. The dose was determined as follows:

**► 800mg Iron carboxymaltose**

→ 16ml Ferinject in 200ml 0.9% NaCl as short infusion over 15 min intravenously

Placebo

The placebo is an unstained 0.9% NaCl solution. A placebo preparation identical in colour to the verum is not available. In order to ensure blindness, the study drug is administered by an external nurse with an eye-protected infusion bag and infusion set.

**4.6 Randomization and Blinding in the Intervention Study**

The participants are randomized to the verum or placebo group in a 1:1 double-blind ratio. Stratification is performed according to the ferritin value at screening (≤25 μg/l or >25 μg/l), gender and the degree of pre-existing fatigue (<4 points versus ≥4 points on the numerical rating scale). Randomisation and stratification is carried out by the nurses of the Clinical Investigation Unit using the computer of the study database.

Since the placebo and the verum preparation differ in colour, the preparation and administration of the infusion must be carried out by an external person who is not otherwise involved in the care of the subjects. This task is performed by nurses from the Clinical Investigation Unit (CIU) of the Inselspital. The nurses of the CIU randomize the participants on the computer, bring the prepared infusion to the blood donation center and administer it. With the help of black colored infusion tubes and by covering the infusion bag with cloths, it is ensured that neither the test person nor the staff of the blood donation center can find out the study arm. Iron carboxymaltose is associated with slightly more frequent side effects compared to placebo^24^. Especially gastrointestinal complaints and general symptoms have been described. However, the extent of these side effects is so small that they will not have any relevant influence on blinding and thus statistical significance.

**4.7 Premature study termination**

The participant can withdraw from the study at any time without giving a reason and withdraw his/her declaration of consent. In this case, no further data will be collected and all blood samples will be destroyed. However, data already collected will be included in the statistical analysis of the study. If the study drug has already been administered, a final medical examination will be performed at the request of the participant.

**4.8 Care of the participants**

In the survey study, the blood donor information and data collection is supported by the nursing staff of the blood donation service. The intervention study is supervised by the study assistant specially employed for the study. She works independently of the blood donor service. She is responsible for the recruitment and information of the volunteers, the care of the participants in the blood donation centre and the completion of the questionnaires. Before the study drug is administered, the donor physician checks and verifies the declaration of consent and answers any additional questions the participant may have about the study.

**5. Selection of study participants**

**5.1 Survey study**

Consecutive blood donors at the Blood Donation Centre Berne (Murtenstrasse 42, 3008 Berne) of the Blood Donation Service of the SRC Berne Ltd. are invited to participate in a survey study on iron deficiency on the occasion of their donation. The donor must agree to an additional blood sample and to answer a questionnaire.

**5.1.1 Inclusion criteria**

- Signed consent form

- Whole blood donors suitable for donation

**5.1.2 Exclusion criteria**

- Active disease or medication associated with fatigue

- Limited ability to understand the consent form

- Limited ability to complete the questionnaire

- Initial donor

**5.2 Intervention study**

All participants in the survey study with a serum ferritin ≤50.0 μg/l who meet the inclusion criteria and have no exclusion criteria will be invited to participate in the intervention study by telephone by the study coordinator.

**5.2.1 Inclusion criteria**

- Participation in the overview study

- Donation suitability

- At least one previous blood donation

- Signed declaration of consent

- Serum ferritin ≤50.0 μg/l

**5.2.2 Exclusion criteria**

- Anemia: Hemoglobin <121 g/l (♀) or <135 g/l (♂)

- Previous intolerance reaction to intravenous iron

- Undergone anaphylactic reactions

- Active systemic infections of any kind

- Known congenital or acquired iron overload (hemochromatosis)

- indications of acute or chronic bleeding, especially gastrointestinal

- Active disease or medication that would explain increased fatigue (at the discretion of the study director/donor physician)

- Active disease or medication that makes intravenous iron replacement not advisable (at the discretion of the study director/donor physician)

- Body weight <50 kg and >85 kg (♀) / >100 kg (♂)

- Age <18 years or >70 years

- Pregnancy and breastfeeding

- Limited ability to complete the questionnaires

- Limited ability to understand the consent form

**6. Measurements**

**6.1 Survey study**

**6.1.1. Questionnaire**

Each participant is asked to complete a questionnaire. This questionnaire consists of 14 questions and evaluates the following topics:

- Previous donor behaviour (2 questions)

- Pre-existing anemia (2 questions)

- Subjectively perceived fatigue (numerical rating scale from 1 - 10)

- Physical performance (numerical rating scale from 1 - 10)

- Concentration ability (numerical rating scale from 1 - 10)

- Loss of drive (numerical rating scale from 1 - 10)

- Depressive mood (Two numerical rating scales from 1 - 10)

- Hair Loss

- Changes in fingernails

- Fatigue caused by blood donation (2 questions)

**6.1.2. Laboratory analyses**

During routine donation preparations, capillary haemoglobin level is determined. A 2.7 ml EDTA tube and a 10 ml serum tube are collected from each participant. The serum ferritin level is then determined in each participant. In a random selection of all participants (one fifth of participating persons), a complete blood count including erythrocyte indices, CRP, soluble transferrin receptor, transferrin saturation, zinc protoporphyrin and serum hepcidin is also determined. Since some of the analyses are performed "en bloc" at a later date, the material is portioned and frozen at -70° C.

**6.2 Intervention study**

**6.2.1. Questionnaires**

The psychometric assessment is carried out with standardised questionnaires validated in German by participants self-report in terms of the following dimensions:

1) Tiredness (fatigue)

2) Health-related quality of life

3) Psychological distress / mood

The questionnaires are handed out to the participants as a booklet. The participant answers the questions independently, and the study coordinator is available in case of any uncertainties. The answering of the questions takes place in the blood donation centre and takes about 30 minutes.

The numerical rating scale from 1 to 10 for the symptom fatigue (main endpoint) was tested in a preliminary trial with 79 healthy blood donors. The median fatigue level was 3, with only 7 of 79 of participants indicating the lowest possible value of 1. Nine of 79 participants quantified their level of fatigue as 2, 29 of 79 as 3, and 24 of 79 indicated a value of 5 or higher. Thus, the test is well suited to measure fatigue and allows almost all subjects to indicate also low levels of fatigue.

At the second visit, an additional numerical rating scale with a range from -10 to +10 is used to determine whether the participant’s fatigue has changed subjectively (secondary endpoint).

Applied tests:

- Perception of fatigue with a numerical rating scale from 1 to 10^5^

- Numerical rating scale from -10 to +10 for the subjectively perceived change in

fatigue (at second study visit only)

- Multi-dimensional fatigue symptom inventory, short form^23^ (MFSI-SF): 30 items

- EUROQol for quality of life^25^ (EQ): 6 items

- Symptom checklist SCL-27^26^ (SCL-27): 27 items

- Jenkins sleep questionnaire^27^ (JSQ): 4 items

**6.2.2. Laboratory analyses**

At both the first and second visit, 10 ml serum and 2.7 ml EDTA blood are collected. The blood count including erythrocyte indices (Advia 120), CRP, serum ferritin, transferrin saturation, soluble transferrin receptor, zinc protoporphyrin and serum hepcidin are determined. Since some of the analyses are performed "en bloc" at a later date, the material is portioned and frozen at -70° C. In women of childbearing age, a urine pregnancy test is carried out before the participant is included in the study.

**7. Safety**

**7.1 Safety profile of the trial drug**

Iron carboxymaltose (Ferinject®) has been approved in Switzerland since 2008. The preparation is intended to be administered in medical practices without special safety equipment. Iron carboxymaltose (Ferinject®) has a very favourable side-effect profile^28^. Most common are reactions at the injection site. Mild headaches, gastrointestinal complaints and skin rashes are also observed. Anaphylactic reactions, as feared with earlier intravenous iron preparations, are a great rarity with Ferinject®. The Blood Donation Centre in Berne would be ideally prepared to treat such complications. The dose was chosen so that no volunteer would suffer from iron overload.

**7.2 Definitions**

**(See also Appendix B, page 13)**

**7.2.1. adverse event (AE)**

For the purposes of this protocol, an adverse event (adverse event = AE) is any occurrence or worsening of a symptom, pathological finding or medical condition during or after administration of the study drug, regardless of whether a relationship with the study drug is suspected. Side effects are evaluated by means of open questions. If available, more detailed and specific questions follow. A physical examination, special laboratory tests or other tests for detection are not routinely performed and are only ordered if the severity or nature of the condition medically requires it.

**7.2.2 Serious adverse event (SAE)**

A serious adverse event (SAE) is any adverse reaction that

- results in death

- is life-threatening

- requires hospitalization

- results in continuing disabilities

- leads to congenital anomalies / birth defects in a child

- is otherwise medically significant (in the opinion of the examiner)

**7.2.3 Adverse drug reaction (ADR)**

An adverse drug reaction (ADR) is any harmful or undesirable reaction to the study drug, if a relationship with the study drug must be suspected. It can be a known adverse reaction or one that has never been described before.

**7.2.4 Suspected unexpected serious adverse reactions (SUSAR)**

A suspected unexpected serious adverse reaction (SUSAR) is any adverse reaction that has already been described but was more serious than described in the medication information.

**7.3 Documentation and evaluation of side effects**

**(See also Appendix B, pages 14 to 16)**

The severity of side effects is classified according to the NCI v.3.0 criteria. All side effects and all therapies administered for their treatment are recorded in the patient documentation and in the eCRF. If side effects are still present at the second study visit, they are tracked until they disappear (by telephone if medically justifiable). SAEs are also listed on a special SAE form. Whether there is a correlation with the study medication is assessed as described in Appendix B, point 4.3.

**7.4 Notification of adverse events and annual safety report**

**(See also Appendix B, pages 17 to 35)**

The Principal Investigator as investigator/sponsor of the study ensures that all SAEs and SUSARs are reported to him/her by the study team, usually on the same day. He ensures that an authorised and competent deputy is always available in case of absence.

Life-threatening and fatal SAEs are immediately reported to the Cantonal Ethics Committee (CEC). The remaining SAEs are summarised in an annual safety report for the attention of the CEC Berne. Serious Adverse Drug Reactions (SARs) and SUSARs are reported to the Cantonal Ethics Committee and Swissmedic in accordance with official guidelines. The exact procedure is described in Appendix B, point 4.5.3.

**7.5 Pregnancy**

Every woman of child-bearing age has to undergo a urine pregnancy test before enrolment. Pregnant women cannot be included in the study. All women of childbearing age are required to prevent conception for at least three weeks. If no permanent (medicinal or mechanical) contraception is used, temporary contraception with a mechanical method (condom) is required.

At the second study visit, specific questions are asked as to whether a pregnancy has become known in the meantime (a pregnancy test is not carried out). If this is the case, the outcome of the pregnancy will be inquired from every woman after she has given birth and the outcome is recorded in the eCRF.

**8. Power analysis and statistics**

The main endpoint of the intervention study is the difference in the mean level of fatigue on the numerical rating scale between the ferinject and placebo groups four to six weeks after infusion of the study drug. Based on empirical values from the literature, a standard deviation of the fatigue values of 2.5 points can be assumed. A mean difference of one point on the scale is considered clinically relevant.

200 subjects per group lead to a power of >95% to detect such a reduction with a two-sided α error of 0.05. For a secondary analysis this number of cases leads to a power of about 85% to detect a statistical interaction of the treatment effect with a subgroup in the order of 1.5 points. The primary stratification criterion will be the ferritin value at baseline (<25 µg/l versus ≥25 µg/l). Secondary stratification criteria will be sex and fatigue at baseline (<4 versus ≥4 on the numerical rating scale).

The mean values on the numerical rating scale will be compared by means of covariance analysis between the groups corrected for baseline value of the numerical rating scale. For stratified analyses we will perform an interaction test using least squares regression models. All analyses will be performed according to the intention-to-treat principle, including all randomized patients in the group to which they were initially randomized. Any missing values are replaced by multiple imputation. In a sensitivity analysis only subjects with complete data are included. All P-values are two-sided. The data analysis is performed under blind conditions, i.e. the statistician in charge will not know which of the two groups analysed received iron and which placebo. All analyses are performed in Stata Release 11 (StataCorp, College Station, TX).

**9. Study specific precautions**

The study drug is generally well tolerated. Higher degree side effects are rare. Special precautions are not indicated after administration of the study medication. When the participants continues to feel well, driving is not prohibited after administration of the study medication. If side effects occur after administration of the study medication, the participants are requested to contact the donor physician at the Blood Donation Centre Bern immediately or, in an emergency, an emergency doctor. After administration of the study medication, the participant is given an information sheet with contact details.

**9.1 Pregnancy**

There is no evidence of embryonic or fetal toxicity of iron carboxymaltose. However, in the absence of data, administration during the first trimester is contraindicated. For this reason, a urine pregnancy test is performed in every woman of childbearing age (menarche to confirmed menopause). If the result is positive, the volunteer cannot be included in the study. Electronic randomisation for the study is only possible after a negative result of the pregnancy test has been entered into the eCRF and validated.

A serum pregnancy test is not practical in the planned study setting. Since the early pregnancy risk of iron carboxymaltose can be considered very low (no pregnancy complications due to iron carboxymaltose were reported two years after market approval) and a urine test, if performed correctly, detects pregnancy in >99% of cases, the safety of a urine pregnancy test is sufficiently guaranteed. It can also be assumed that urine pregnancy tests are generally carried out in medical practice before Ferinject® is administered.

The medication information approved by Swissmedic does not give any recommendation as to how long after the administration of the medication a conception must be avoided. In everyday clinical practice, therefore, contraception is not necessarily ordered after administration of Ferinject®. Iron carboxymaltose is very rapidly absorbed into the macrophages of the reticulo-endothelial system, where it is broken down into iron hydroxide and sugar components. The elimination half-life in blood is 7 to 12 hours^28^. After this time, iron is bound to transferrin and ferritin, which prevents toxic effects.

Based on the above considerations, study participants of childbearing age must commit themselves to a minimum of three weeks of anti-conception after administration of the study medication. If no permanent anti-conception (mechanical or medication-based) is performed, mechanical methods (e.g. condoms) must be used for three weeks. No anti-conception measures are indicated for female partners of male study participants.

**10. Duties of the auditor**

**10.1 Compliance with legal requirements**

The study will be conducted in accordance with Good Clinical Practice (GCP) requirements. Any changes to the protocol will be submitted to the Cantonal Ethics Committee of Berne and Swissmedic for approval before they come into force. The Principal Investigator, who is also the sponsor of the study, is responsible for training all staff involved in the study. He ensures compliance with the study protocol, GCP guidelines and legal requirements.

**10.2 Reporting**

**(See also Appendix B)**

The Principal Investigator, who is also the sponsor of the study, is responsible for the timely reporting of serious adverse reactions (SAEs, SARs, SUSARs) to the Cantonal Ethics Committee and to Swissmedic. The details are regulated in Appendix B. A safety report is written up once a year. In a final report, the most important study results are summarised by means of suitable graphs and tables. The report is unbiased and objective. The reports are submitted to both the Cantonal Ethics Committee of Berne and Swissmedic within the prescribed time limits.

**10.3 Insurance**

All study participants are insured under the group insurance contract between the Inselspital Bern and AXA-Winterthur Insurance for losses incurred during participation in the study.

**11. Ethical considerations**

**11.1 Survey study**

In the survey study, two additional blood tubes are taken from the volunteer during a whole blood donation. In addition, the volunteer has to fill out a questionnaire, which takes about 15 minutes. Apart from this, the Participant does not suffer any disadvantages. The anonymity of the data is guaranteed. The findings of the survey study extend our knowledge of the extent of the iron deficiency problem in regular blood donors and thus benefit all blood donors.

Since the participants do not suffer from anaemia according to the inclusion criteria, there is no need for action from a medical point of view. It is therefore not planned to send the results of the iron status to every study participant. However, the results will be provided at the next blood donation or upon request by telephone.

**11.2 Intervention study**

Iron carboxymaltose (Ferinject®) has a very favourable side-effect profile^28^, the most common being injection site reactions. Mild headaches, gastrointestinal complaints and skin rashes are also observed. Anaphylactic reactions, as feared with earlier intravenous iron preparations, are a great rarity with Ferinject®. The blood donation centre in Berne is also well prepared to treat such complications. The dose of iron carboxymaltose was selected so that, although the iron stores are sufficiently filled in each participant, iron overload with long-term toxic damage cannot occur at the same time. It is not to be expected mathematically that the serum ferritin will exceed the upper laboratory norm after reaching a state of equilibrium. In summary, the potential risks of the study drug are very low.

Half of participants will receive iron substitution, the other half placebo only (0.9% NaCl). Although it is now common practice for many doctors to substitute iron deficiency without anaemia, the risk of iron deficiency is very low. But this is not based on scientific data. From the point of view of evidence-based medicine, there is therefore no proven advantage neither for a person in the placebo group nor for one in the verum group.

After completion of the intervention study, each participant is informed in writing whether he or she has received the iron supplement or the placebo. In addition, the laboratory results of the first study visit and the ferritin value will be sent to the participants at the final visit. This information allows the treating family doctor to continue to treat the patient correctly.

The study promises very relevant results both for patients and healthy blood donors with iron deficiency without anaemia. The lower limit of the ferritin level above which iron must be substituted could be more precisely defined, thus enabling unnecessary iron infusions to be avoided in the future. If adverse effects of iron deficiency on blood donors can be demonstrated, blood donation procedures would need to be adapted. This would benefit all blood donors in the long term. Based on all of these considerations, the general medical benefit of the study justifies the very low risk for the individual study participant.

**12. Quality assurance**

**12.1 Monitoring**

**(See Appendix A: Monitoring Plan)**

The monitoring of the study is carried out by the Clinical Trials Unit (CTU) of the Inselspital in compliance with GCP guidelines. There will be no CTU staff involved in the monitoring process who are otherwise involved in the study. The exact procedure is defined in Appendix A (Monitoring Plan).

**12.2 Inspections**

The Cantonal Ethics Committee of Berne and the competent authorities are granted access for audits and inspections. Access to all original data and the electronic database is guaranteed.

**12.3 Handling of data**

**(See also Appendix B, pages 30+31)**

Each participant will fill out a questionnaire set twice. Additional medical information will be entered in a standardized form. This corresponds to the source document. All data will be entered in Webspirit. Webspirit is a web-based clinical study software for electronic data entry. The database performs validity checks to detect errors or suspicious outliers. It contains integrated monitoring functions including the generation of queries. The database is centrally managed and maintained by CTU Bern.

**12.3.1 Case Report Forms**

All data entered into the electronic "Case report form" (eCRF) must be stored in the source document. The "Case report form" is not considered a "source document". All data collected and measures taken as prescribed by the protocol are entered in the eCRF. The data are entered into the eCRF by the study assistant. The entries must be complete, accurate and punctual (within one week) after the study visit.

The Principal Investigator or a deputy authorized by him/her will check the entries randomly for accuracy and compliance with the protocol and sign the eCRF if it is complete. After prior notice, the compliance of the source data and the eCRF entries may be checked by the monitor or the competent authorities. The Principal Investigator guarantees the identity of the raw data and the entries in the eCRF.

**12.3.2 Archiving**

The final case report forms are printed out and stored in a study folder together with the other documents (e.g. the protocol) in a safe, sealed place. The Principal Investigator is responsible for long-term archiving.

**12.3.3 Electronic database**

The electronic database infrastructure is provided by the CTU Bern. Entries can be made web-based on local computers of the participating institutions. The study data is stored in a relational SQL database using the Webspirit system of CTU Bern.

The database servers are located in a locked, protected room. Only the system administrators have access to the server. A back-up is created daily and is stored in a different building. A second backup is stored on a server in the Institute for Social and Preventive Medicine. Authorised users can access the database by means of a personal password.

**12.3.4 Data validation**

A multi-level validation plan guarantees the correctness and consistency of the data. The data can only be saved after a check for completeness and plausibility. Data entry is done via the Internet with masks that check the consistency of the data. Data is also checked for consistency with previously entered data. Any subsequent adjustment of the data is recorded electronically. The identity of the participant is guaranteed by a case ID. This ID is automatically assigned to the participant by the database. There is no logical connection between the case ID and the name of the participant. Data exchange takes place exclusively using the case ID.

**12.3.5 Data analysis and archiving**

The data is transferred to statistical software for evaluation. At this point, the database is transferred to an archive form that cannot be changed later. All study data in paper form are stored in steel containers for 10 years under the responsibility of the Principal Investigator. The SQ database is also retained by the CTU for 10 years. CTU Bern will retain interim and final reports in both electronic and paper form.

**12.4 Handling blood samples**

Not all of the intended analyses are performed immediately. Various analyses will only be carried out at a later date. For this purpose, serum is aliquoted and frozen at -70° C. Storage takes place in the routine laboratory of the Blood Donation Service Bern at Murtenstrasse 133, 3008 Bern. The freezer is always locked. All samples are destroyed at the latest one year after completion of the study. No analyses are performed that are not provided for in the protocol.

**13. Additional information for the notification (Swissmedic)**

**13.1 Drug Accountability**

The study drug is delivered directly to the Clinical Investigation Unit (CIU) of the Inselspital, where it is locked, inaccessible to unauthorized persons and stored at room temperature (+4° C to 25° C). The receipt of the medication is noted with lot number and expiration date on the form "Medication deliveries" and signed by a CIU nurse.

Each withdrawal of Ferinject® ampoules from the appropriately labelled warehouse in the CIU is recorded on the ISUB study's "Medication Log Form" and signed by the appropriate CIU nurse. The name and date of birth of the participant, the number and size of the used drug vials, the lot number and the expiration date are recorded.

**13.2 Recording compliance**

The intravenous administration of the study drug is carried out under the supervision of a CIU nurse on the day of admission to the study (i.e. immediately after signing the consent form). Problems with malcompliance are therefore not to be expected. If for any reason the infusion cannot be administered, the CIU nurse will note this on the Case Report Form, the eCRF, and the study drug log form

**13.3 Labelling of the IMP**

The commercial preparation Ferinject® is supplied by Vifor Pharma SA in the usual packaging and normally labelled. The packaging and vials contain a lot number and the expiration date.

The infusion is prepared by a CIU nurse according to computer randomization. This is done on the premises of the CIU under sterile conditions. The infusion bag (verum or placebo) is labelled with a printed label containing the subject number, the study name (ISUB study), the principal investigator/sponsor (University Department of Haematology, Inselspital Bern), the date and time of manufacture. Randomisation (verum versus placebo) is not indicated on the label.

The infusion must be made immediately after preparation (maximum of 120 minutes). To transport the prepared infusion from CIU to the blood donation centre (about 200 metres on foot on public roads), the infusion bag is packed in a clean bag and the whole infusion is transported in a tightly sealed container. The infusion is always under the supervision of the CIU nurse who will administer the infusion at the blood donation centre.

**13.4 Publication of the results**

The results of the study will be published in at least one scientific journal. All formal presentations and publications of data from this study are made as joint communications of all scientific staff. For the first publication, Dr. P. Keller is the first author and Dr. S. Fontana the last author. Further co-authorships are determined according to the scientific contribution to the study and should be based on the unanimous agreement of all scientific collaborators. Further analysis of data and publication of results (scientific articles, lectures) may only be performed with the joint consent of Dr. P. Keller and Dr. S. Fontana.

14. Literature

1. Andrews NC. Disorders of iron metabolism. N Engl J Med. 1999;341:1986-1995.

2. Brownlie Tt, Utermohlen V, Hinton PS, Giordano C, Haas JD. Marginal iron deficiency without anemia impairs aerobic adaptation among previously untrained women. Am J Clin Nutr. 2002;75:734-742.

3. Bruner AB, Joffe A, Duggan AK, Casella JF, Brandt J. Randomised study of cognitive effects of iron supplementation in non-anaemic iron-deficient adolescent girls. Lancet. 1996;348:992-996.

4. Patterson AJ, Brown WJ, Roberts DC. Dietary and supplement treatment of iron deficiency results in improvements in general health and fatigue in Australian women of childbearing age. J Am Coll Nutr. 2001;20:337-342.

5. Verdon F, Burnand B, Stubi CL, et al. Iron supplementation for unexplained fatigue in non-anaemic women: double blind randomised placebo controlled trial. Bmj. 2003;326:1124.

6. Der Iron-Code. Coop-Zeitung vom 6 November 2007; #45.

7. Skikne B, Lynch S, Borek D, Cook J. Iron and blood donation. Clin Haematol. 1984;13:271-287.

8. Newman B. Iron depletion by whole-blood donation harms menstruating females: the current whole-blood-collection paradigm needs to be changed. Transfusion. 2006;46:1667-1681.

9. Raftos J, Schuller M, Lovric VA. Iron stores assessed in blood donors by hematofluorometry. Transfusion. 1983;23:226-228.

10. Simon TL, Garry PJ, Hooper EM. Iron stores in blood donors. Jama. 1981;245:2038-2043.

11. Pedersen NS, Morling N. Iron stores in blood donors evaluated by serum ferritin. Scand J Haematol. 1978;20:70-76.

12. Finch CA, Cook JD, Labbe RF, Culala M. Effect of blood donation on iron stores as evaluated by serum ferritin. Blood. 1977;50:441-447.

13. Milman N, Sondergaard M. Iron stores in male blood donors evaluated by serum ferritin. Transfusion. 1984;24:464-468.

14. Frey BM, Rüfer A, Hardegger K, et al. Iron Balance Remains Stable in Life-Long Blood Donors (Poster DGTI 2007). Transfus Med Hemother. 2007;34 (Suppl 1):69.

15. Mast AE, Foster TM, Pinder HL, et al. Behavioral, biochemical, and genetic analysis of iron metabolism in high-intensity blood donors. Transfusion. 2008;48:2197-2204.

16. Schorer G, Brittenham G, Darnuzer R, Westerman M, Frey BM. Sequential blood donation decreases total body iron stores and serum hepcidin (Poster SGIM/SGH 2009). Schweiz Med Forum. 2009;9 (Suppl 46):79 S.

17. Rosvik AS, Hervig T, Wentzel-Larsen T, Ulvik RJ. Iron status in Norwegian blood donors: comparison of iron status in new blood donors registered in 1993-1997 and in 2005-2006. Vox Sang. 2009;96:49-55.

18. Ochmann O, Hardegger K, Frey B. Iron Balance in Young Blood Donors: Is the Latent Iron Deficiency Syndrome (LIDS) Universal? (Poster DGTI 2007). Transfus Med Hemother. 2007;34 (Suppl 1):71.

19. Radtke H, Tegtmeier J, Rocker L, Salama A, Kiesewetter H. Daily doses of 20 mg of elemental iron compensate for iron loss in regular blood donors: a randomized, double-blind, placebo-controlled study. Transfusion. 2004;44:1427-1432.

20. Maghsudlu M, Nasizadeh S, Toogeh GR, Zandieh T, Parandoush S, Rezayani M. Short-term ferrous sulfate supplementation in female blood donors. Transfusion. 2008;48:1192-1197.

21. Magnussen K, Bork N, Asmussen L. The effect of a standardized protocol for iron supplementation to blood donors low in hemoglobin concentration. Transfusion. 2008;48:749-754.

22. Pedrazzini B, Waldvogel S, Cornuz J, et al. The impact of iron supplementation efficiency in female blood donors with a decreased ferritin level and no anaemia. Rationale and design of a randomised controlled trial: a study protocol. Trials. 2009;10:4.

23. Stein KD, Jacobsen PB, Blanchard CM, Thors C. Further validation of the multidimensional fatigue symptom inventory-short form. J Pain Symptom Manage. 2004;27:14-23.

24. Bailie GR, Mason NA, Valaoras TG. Safety and tolerability of intravenous ferric carboxymaltose in patients with iron deficiency anemia. Hemodial Int;14:47-54.

25. Rabin R, de Charro F. EQ-5D: a measure of health status from the EuroQol Group. Ann Med. 2001;33:337-343.

26. Hardt J, Egle UT, Brahler E. [The symptom checklist-27 in Germany]. Psychother Psychosom Med Psychol. 2006;56:276-284.

27. Jenkins CD, Stanton BA, Niemcryk SJ, Rose RM. A scale for the estimation of sleep problems in clinical research. J Clin Epidemiol. 1988;41:313-321.

28. Ferinject Fachinformation. Arzneimittel-Kompendium der Schweiz. 2010; Band 1: Seiten 1700 -1702.
